# Concatenation fails to describe the anomalous radiation of giant cockroaches (Blattodea: Blaberidae) despite moderate to low discordance

**DOI:** 10.1186/s12862-025-02409-4

**Published:** 2025-07-21

**Authors:** Dominic A. Evangelista, Michael A. Gilchrist, Frédéric Legendre, Brian O’Meara

**Affiliations:** 1 Department of Ecology and Evolutionary Biology, The University of Tennessee, Dabney Hall, 1416 Circle Dr, Knoxville, TN 37996 USA; 2 Institut de Systématique, Evolution, Biodiversité (ISYEB), Muséum National d’Histoire Naturelle, CNRS, Sorbonne Université, EPHE-PSL, UA, 57 Rue Cuvier, CP50, Paris, FR 75005 USA; 3 National Institute for Mathematical and Biological Synthesis, Knoxville, TN 37996 USA; 4 Entomology Department, School of Integrative Biology, University of Illinois, 550 S Goodwin Ave. Room 465 Morrill Hall, Urbana-Champaign, IL 61801 USA

**Keywords:** Rapid radiation, Congruence, Discordance, Coalescence, Incomplete lineage sorting, Tree error, Mutation selection, Nested codon model

## Abstract

**Background:**

Patterns of discordance between gene trees and the species trees they reside in are crucial to the coalescent vs. concatenation debate and may be key to resolving rapid radiations. However, errors in gene trees complicate the issue as topological errors can cause gene trees to appear erroneously discordant with the species tree. In this study, we evaluate the prevalence of discordance between gene trees and their species tree using an empirical dataset for a clade with a rapid radiation (Blaberidae). One key advance of our study is the use of complex, computationally intensive, selection-based codon models (FMutSel0 and SelAC) to identify the maximum likelihood gene tree. Our main hypothesis predicted that, if there are two competing topologies for a particular gene tree, then the one that is less discordant with the species tree will have less systematic error.

**Results:**

Our experimental framework failed to show evidence for this, but only when discordance was measured in reference to a concatenation topology. In follow-up tests we see that the best candidate gene set yielded a coalescent species tree that was less discordant with gene trees.

**Conclusions:**

We conclude from these tests that, although the frequency of discordance is on the low end of what is predicted by a range of modelling strategies, it is still extremely common overall and must be accounted for in order to achieve a biologically realistic outcome. These results allow us to support other relationships among blaberid cockroaches that were previously in flux as they now demonstrate molecular and morphological congruence. We suggest a few key improvements to the Blaberidae phylogeny, including identification of an anomaly zone spanning 10 backbone nodes and 6 additional nodes.

**Supplementary Information:**

The online version contains supplementary material available at 10.1186/s12862-025-02409-4.

## Background

Debates over the relative merits of phylogenetic species tree (ST) inference using concatenation or the multi-species coalescent are ongoing [20, 29, 67]. Edwards, et al. [20] argues that concatenation represents a “special case” of coalescence where all gene tree (GT) topologies are presumed to be the same. Concatenated and coalescent phylogenetic inference usually yield similar topologies [4, 20, 46], but see [32, 54]. This suggests that genes commonly share the same evolutionary history. Indeed, empirical studies have shown that better supported GT topologies tend to be less discordant [6, 76], incomplete lineage sorting may not be common [27], and in that case concatenation will be more accurate than coalescence [11]. In the current study, we investigate how gene tree-to-species tree (GTST) discordance varies with evolutionary model complexity using an empirical dataset of Giant Cockroaches (Blattodea: Blaberidae). Blaberidae is well-studied in numerous aspects [15] but their phylogeny has proven difficult to infer due to a rapid radiation [21, 47, 91].

The biological processes contributing to GTST discordance (i.e., incomplete lineage sorting, hybridization, and lateral gene transfer; [36, 84]) can be accounted for through coalescent theory (e.g., [52]) but are ignored in concatenation tree inference. Since hybridization and lateral gene transfer events are thought to be uncommon [12, 53], many studies presume incomplete lineage sorting is responsible for all discordance between GTs and the ST (e.g., [11].

GT error is also expected to contribute to GTST discordance [27]. GT error can derive from unaccounted biological processes (systematic error), and low signal introducing high stochasticity into the analysis (estimation error). Inference of a single GT necessarily suffers from high estimation error, since single genes are only a few hundred or thousand nucleotides long. Systematic error can also be present when modelling mutational variance within a GT is difficult [35, 40, 59]. Evolutionary history is complex and may only be accounted for by models that reflect that complexity [13, 85]. Estimation error compounds systematic error because the limited character information in genes amplifies the effect of small errors in the modelling of sequence evolution [27]. Other studies have shown that leveraging informed prior distributions in Bayesian inference may improve GT estimation, as long as MCMC chains properly converge [27], and that some reliability metrics may be good proxies for GT accuracy [68].

Whatever errors occur in GT inference affect downstream coalescent analyses to varying degrees. The multi-species coalescent model assumes that loci are independent and evolve according to random processes [14]. However, these assumptions are known to be violated throughout the tree of life [13, 19]. As such, even when ST estimation error is low due to a large sample of GTs, systematic error could be abundant if care isn’t taken in model and gene choice [59]. Considering this, GT error may be even more important to consider, since errors at the GT level could accumulate with error at the ST level. Although when analyzing single nucleotide polymorphism data there are ST estimation methods that bypass GT error [70], or GTs estimation [8] entirely. In other use cases, some coalescent ST approaches account for uncertainty in GTs [28, 63, 90], but most methods do not [42, 46, 51, 83]. In either of these cases, improved accuracy in GTs would likely lead to more accurate STs since coalescent methods can be highly sensitive to small changes in GTs [73, 90] or rogue GTs [64]. All the same, coalescence may be less vulnerable to these errors than concatenation [73], which assumes completely identical GTs.

In all, our study aims to evaluate the implications of GTST discordance at both the level of GT choice, and ST inference. First, we investigate what level of GTST discordance is present when we attempt to minimize GT error based on the assumption that more complex, and biologically realistic, models will better model nucleotide substitutions and extract more signal from single loci. To that end, we utilize an experimental framework examining how selection-based codon models (SelAC; [5], FMutSel0; [96]), and the general time-reversible nucleotide model [82] choose among protein coding GT topologies with differing levels of discordance. We hypothesize that these models will more often prefer the GT topology less discordant with the ST. Thus, we predict that much GTST discordance is error [76]; see above for explanation of the type of error), which explains why concatenation often yields robust STs. Second, we compare how different GT sets, analyzed in ASTRAL, affect coalescent STs. The goal is to both identify the best ST for our empirical dataset and determine how various criteria of choosing GTs (and levels of GTST discordance) perform.

One reason codon models are thought to improve phylogenetic inference over nucleotide models is because they are believed to more accurately reflect the evolutionary forces acting on protein coding genes [2, 16, 31, 72, 89]. Modelling codon evolution is also thought to be more effective than modelling amino-acids since evolution occurs at the codon level [2]. The codon-based models SelAC and FMutSel0 are similar in that they include selection to model mutational evolutionary history, but differ in their complexity [5]. FMutSel0 is the simpler of the two codon models because it uses a single parameter, omega, to model selection. While omega is commonly interpreted as representing stabilizing (omega < 1) versus diversifying selection (omega > 1), conceptually the term is more consistent with models of negative and positive frequency dependent selection, respectively [5]. In contrast, SelAC explicitly models stabilizing selection for an optimum sequence of amino acids based on their physico-chemical properties. Drawing on arguments about cell energetics, SelAC also assumes the strength of stabilizing selection scales with the average protein production rate of the gene (i.e. gene expression [18]). As a result, rather than using a single substitution matrix, SelAC employs 20 different families of matrices which are scaled by a site-specific selection term for the optimal physico-chemical properties and gene expression. Despite its high degree of complexity, SelAC can generate and scale these matrix families using a relatively small number of parameters (~ 40) that are shared across all genes plus one expression-level parameter for every gene.

**Fig. 1:**
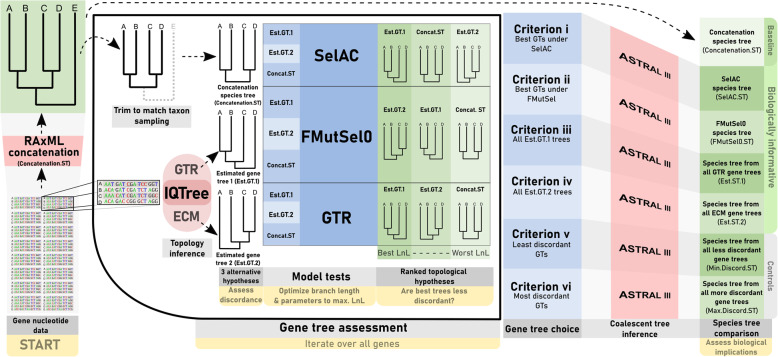
A schematic diagram of the workflow for testing the abundance of gene tree (GT) discordance under three models, and downstream effects in the species tree (ST). “Est.GT.1” refers to GT topologies inferred by GTR. “Est.GT.2” refers to GT topologies inferred by the Empirical Codon Model (ECM). Concat.ST is RAxML concatenation topology trimmed to match the taxon sampling of the GT (i.e., a hypothetical GT for a ST with no GT-ST discordance)

A major motivation for our work is the fact that improving characterization of GTST discordance can improve our ability to resolve rapid radiations [30]. Resolving radiations is difficult because of low phylogenetic information from short periods of time and the increased effect of incomplete lineage sorting on short branches [14, 30, 54]. Our approach (Fig. 1) attempts to improve resolution of rapid speciation in two ways: by using two codon models (SelAC and FMutSel0) that, in theory, are more biologically plausible than simpler models (e.g., GTR, Empirical Codon Model/ECM; [71] and by using a coalescent method [56]. Coalescent methods account for incomplete lineage sorting, which can confound resolution on short internodes if not accounted for. In short, we hope to reduce systematic error at the GT and ST level through more realistic substitution models and use of the multi-species coalescent.

A schematic diagram of the workflow for testing the abundance of gene tree (GT) discordance under three models, and downstream effects in the species tree (ST). “Est.GT.1” refers to GT topologies inferred by GTR. “Est.GT.2” refers to GT topologies inferred by the Empirical Codon Model (ECM). Concat.ST is RAxML concatenation topology trimmed to match the taxon sampling of the GT (i.e., a hypothetical GT for a ST with no GT-ST discordance).

We apply the approaches discussed above on a challenging biological dataset from the cockroach family Blaberidae, whose major lineages diversified 100 Ma in a period of only 30–40 Myr. [26, 48]. Evangelista, et al. [25] illustrated that most subfamilies in Blaberidae have not been placed consistently. Yet, even some of the consistent relationships are not supported by more recent analyses [21, 23, 24, 47, 91]. Some specific examples are as follows: which lineage is recovered as sister to the remaining Blaberidae is inconsistent and highly dependent on taxon sampling [24, 91, 97]; the origins of Panchlorinae and Neotropical Epilamprinae as being ancient or more recent is under contention [7, 23, 24, 47], taxa with many morphologically derived traits like *Diploptera punctata*, *Pycnoscelus* spp., and *Aptera fusca* also have no consistent placement across phylogenies [21, 23, 24, 26, 47], some groups (e.g., Paranauphoetinae, Panesthiinae and Perisphaerinae in [1] or Blaberinae and Zetoborinae in [33]) have morphological hypotheses for their relationships that have not been strongly supported by molecular data [7, 23, 24, 47], inconsistent topologies have prevented interpretation of interesting phenotypic diversity [23, 24, 39, 47], recent studies still recover largely incongruent topologies even when they have strong taxon sampling [21–23, 47, 91].

## Methods

All genetic data were taken from Evangelista et al. [23]. From their 265 protein-coding loci, a subset of 100 loci were chosen based on minimizing locus rate heterogeneity (calculated in that study). Each alignment was further reduced to an ingroup sampling of 42 Blaberidae species and 14 other Dictyopteran outgroups to allow for maximal data completeness.

### Species trees and gene trees

A concatenation ST (Concatenation.ST) was taken from Evangelista et al. [23]. Briefly, GTR and GTR + G were applied in a partitioned analysis of a concatenated alignment of 265 loci in RAxML [78] with a single topology assumed for all partitions. Partitioning scheme and model choice were optimized in PartitionFinder 2 [45]. This was subsequently used as an approximate ST topology. As mentioned previously, concatenation methods are often largely in agreement with the best coalescent ST [4, 20, 46], and concatenation is thought to be a better initial estimate of the ST than naïve coalescent trees [75]. We therefore used this concatenation ST as a baseline estimate of the ST to which we would compare each gene tree (GT) and any subsequent coalescent STs. However, as a concatenation tree it assumes that all GTs share the same evolutionary history. Compared to our further analyses below, Concatenation.ST was inferred using nearly ten times more nucleotide data, and almost three times more taxa overall. In the below analyses, seven tips were removed from the ingroup (Blaberidae) due to low data coverage (*Panchlora stolata*, *P. nivea-1*, *P. nivea-2*, *Achroblatta luteola*, *Achroblatta* sp., *Lanxoblatta* sp., *Paranauphoeta discoidalis*).

The tips of the concatenation topology were trimmed [62] to match the taxon sampling of the GTs and STs. Two GTs were then inferred from each locus in IQ-TREE [60], a program that is fast and produces reliable GTs with fragmentary data [6]. The first was inferred using the GTR model (“estimated topology 1”/“Est.GT.1”) with a median-approximated gamma distribution and 4 rate categories (G4), optimized nucleotide frequencies (FO), and the thorough “allnni” search option. The second GT was inferred with the Empirical Codon Model [71], “estimated topology 2”/“Est.GT.2”), G4, and a nucleotide frequency model determined by IQ-TREE’s built-in model finder [40] under the AICc [81] optimality criterion (see supplementary material S1.1 for further details on model choice). We consider all Est.GT.1 as being in the same “model family” since they share substitution models. Similarly, we consider all Est.GT2 as being in their own “model family”. Rate heterogeneity was modelled using the gamma distribution (four rate categories) but with a mean-approximation method. For a given gene, all three topologies were identically rooted with an outgroup taxon. However, the rooting taxon differed among genes (*Metallyticus splendidus*, a solumblattodean, or *Ectoneura* sp.) because of different taxon sampling per gene.

The Robinson-Foulds (RF; [65]) and the path distance [44] among all topologies (Concatenation.ST, Est.GT.1, and Est.GT.2) for each locus were measured using *treedist* in Phangorn [69]. RF distance is the more intuitive metric as it gives a measure of the number of clades shared between trees. However, when even one rogue taxon is present it can give very high distances, which is undesirable. The path distance metric is much less sensitive to rogue taxa but is perhaps a less biologically meaningful descriptor of tree differences [44]. The path distance [80] is the sum of differences in minimal species pair path lengths between trees [44]. All distances were greater than 0, indicating that all three alternative trees for each gene were distinct from one another. Yet, since RF and path distances compare different aspects of tree shape, a given tree can be the more discordant tree under one metric but the less discordant tree under the other. To ensure we were unambiguously ranking alternative GTs for a single gene as “more” or “less” discordant with respect to the ST, we only retained a genes if both RF and path distances yielded the same result on which GT was most discordant to the ST (33% of cases).

### Model tests on gene trees

The experimental design described here integrates three elements: GTST discordance, GT error, and the efficacy of complex codon models. Discordance is the independent variable – the three alternative trees for each locus (Concatenation.ST, Est.GT.1, and Est.GT.2) each have a known amount of GTST discordance with Concatenation.ST (mean RF distance = 61; mean path distance = 112) and they are all distinct from each other (mean RF distance = 45; mean path distance = 93). Concatenation.ST has a discordance of 0 by definition. The other two have different magnitudes of discordance to Concatenation.ST, so one will always be more discordant, and the other will always be less discordant. GT error is the dependent variable, but we are measuring it using lnL as a proxy. As such, the type of error we are measuring is a combination of systematic and estimation error. It is systematic error because, the lnL is indicative of a better fit of the data to the evolutionary model, but it is also estimation error because we assume that better model fit means that the “true” signal in the gene alignment is better captured by the model. Our using lnL in this way is possible because the data (taxa and nucleotide sites) are identical for a given comparison, and the only thing varying is the evolutionary model and the associated branch length optimization parameters. The GT with the best lnL from a single model is assumed to have the lowest error given the parameters of that model. This assumption derives from the third element: the complex codon models (FmutSel0, SelAC). We assume that the complex codon models will identify the tree with the least error as the maximum lnL tree. Our hypothesis predicts that the maximum lnL GT will have lower discordance to the ST than the alternative GT. Thus, the complex codon models serve only as a method to choose between alternative GTs. It would be possible, although computationally burdensome, to allow the complex codon models to freely search GT space, deriving their own hypotheses about the GT relationships. While this approach could also test the above hypothesis, and would benefit ST inference by inferring a more robust ST, it has some other drawbacks. First, it is less straightforward from a hypothesis testing perspective (no specific alternative hypotheses can be eliminated). Also, it would rely even more heavily on the assumption listed above. Given the immensity of total tree-space, it is much less likely for two complex models (e.g., FMutSel0 and SelAC) to converge on the same GT topology compared to our approach, which limits the search space to just three alternatives. As mentioned above, the latter approach is perhaps not computational feasible. As discussed below, optimizing parameters for single genes with SelAC and FMutSel0 takes a huge amount of CPU resources, even when the sister relationships are constant.

Preliminary tests (Supplementary material S2.2) showed that FMutSel0 tended to yield better lnLs for GTs inferred through GTR (Table S.2.2.1) and GTs that were less discordant to Concatenation.ST. In other words, the experiment was not controlled because lnLs from FMutSel0 seemed to be biased towards GTs inferred under GTR. To correct for this bias, we chose 40 loci where the least discordant GTs occurred among Est.GT.1 (GTR) half of the time and Est.GT.2 (Empirical Codon Model) half of the time. These loci had an average occupancy of 44 species per alignment (min. 34, max. 50).

Branch lengths for each of the three trees for each gene were optimized again using the SelAC software package [5]. We fit: GTR + G4 + FO, SelAC + GTR + G4 + FO + AAO, and FMutSel + GTR + G4 + FO + AAO. In both cases, estimated branch lengths from IQ-TREE were used as starting values and optimization chains were run for four sets of initial conditions (Table S1.1.1). Other parameters used were: max.tol.edges = 1.4 and tol.step = 2.3, parallelized over two processors (see example R code in Supplementary Data). Initially, four independent starts were done for all three model schemes. Within these four optimizations, GTR converged on the same lnL (± 1 lnL unit) 100% of the time (360 total optimizations). We assume that if two or more optimizations yielded lnLs within 1 lnL unit of each other that likelihood had been maximized. In these initial runs, the difference in lnL between the independent runs was an order of magnitude less than the average difference in lnL between treatments (trees). This suggests that even if the maximum likelihood peak in parameter space was not found, that an incomplete search could often yield the same result. However, FMutSel0 and SelAC did not converge on the same (± 1 lnL unit) lnL in most preliminary runs, so we ran 8 more optimizations (Table S1.1.1). The best log-likelihood (lnL) of all independent optimizations was chosen as the preferred GT for a given model-GT pair. Thus, we optimized FMutSel0 and SelAC 12 times on three topologies over 40 genes (1560 total optimizations). In cumulative CPU time (assuming each run was perfectly parallelized over two cores) this took 1.4 years for GTR, 12.6 years for FMutSel0, 12.2 years for SelAC for a total of 26.2 years. The median elapsed wall-time for a single GT optimization (in hours) was 28 for GTR, 284 for FMutSel0, and 295 for SelAC. Even with this run time, we estimate that the maximum likelihood GT was only found for 88% (FMutSel0) and 91% (SelAC) of loci. In the remaining 10% of GTs, we assume that the relative lnLs among treatments would be representative of the final result if the model space had been searched more thoroughly (see above for rationale.) We considered more run time to not be feasible,this does induce a potential bias, but against the selection-based codon models, thus conservative for our hypotheses.

If the topology with the best lnL under a given model (i.e., SelAC, FMutSel0, GTR) was also the estimated GT with the lowest discordance, as determined by both RF and path distances, the trial was assigned a 1, otherwise a 0. Z-tests were used to assess if the models identified the least discordant GT more often than expected from randomness, and if the number of GTs chosen was random with respect to the model family of the GT (i.e., Est.GT.1/GTR or Est.GT.2/Empirical Codon Model). A test was also done to see if lnLs were skewed for GTs of a certain model family or for GTs more discordant to the concatenation ST. For the former, the lnL of Est.GT.1 was subtracted from that of Est.GT.2 and we compared the median of the distribution to the null expectation of a randomized sample of ∆lnLs with a Kolmogorov-Smirnoff test. The randomized sample was generated by pooling all lnLs for Est.GT.1 and Est.GT.2 from all models and drawing 4000 at random, with replacement. The same was done for the difference between the lnL of the least discordant estimated GT and the most discordant. Finally, we examined whether the difference in lnLs (∆lnL) between Est.GT.1 and Est.GT.2 depended on the magnitude of hypothesis distinctness. We did this in two ways. First, by looking for correlations between Est.GT.1 to Est.GT.2 RF distance and ∆lnL. Second, by looking for correlations between the difference between each Est.GT’s RF distance to Concatenation.ST and ∆lnL. These analyses were done with a linear regression. All type-I error thresholds were predefined at 0.05 and calculations were done in Mathematica 13 [93].

The above tests were done in reference to Concatenation.ST as baseline, an experimental design choice made *apriori*. Since concatenation is known to be incorrect in the presence of extremely high incomplete lineage sorting, we repeated some of these tests with a coalescent ST. For this we used a naïve coalescent topology (Naïve.Coal.ST) from Evangelista et al. [21], which utilized on 1183 gene trees analyzed in ASTRAL III [56]. We repeated all the above except the second round of GT optimizations using complex codon models and the follow up tests of correlations between lnL’s and discordance.

### Testing the implications on the species tree

The downstream implications of the choice of GT topologies were tested through a coalescent ST inference in ASTRAL-III [56]. ASTRAL-III is an appropriate software because it is consistent with the coalescent process, it allows GTs to have different taxon sampling, and does not co-estimate the GT and ST. Four test STs were inferred with ASTRAL-III from the following sets of GTs: all estimated GT 1 topologies (Est.ST.1), all estimated GT 2 topologies (Est.ST.2), all GTs chosen as optimal by SelAC (SelAC.ST), and all chosen as optimal by FMutSel0 (FMutSel0.ST). These were also compared to the phylogeny inferred from: the larger concatenated dataset of 265 genes (Concatenation.ST,the baseline), the most discordant trees (Max.Discord.ST) and the least discordant ones (Min.Discord.ST).

The model tests on the GTs served as a direct test on if GTST discordance was preferable to certain models, while this ST stage offers a framework for exploring the implications of GTST discordance on ST topologies. To approach this, we wanted to better understand the similarities of the test STs (SelAC.ST, FMutSel0, Est.ST.1, Est.ST2) and the three discordance-based STs (Concatenation.ST, Max.Discord.ST, Min.Discord.ST). For instance, if the distance between a test ST and the Concatenation.ST is low, it would imply that GTST discordance has little effect on the ST topology. If the distance between a test ST and the Min.Discord.ST is low, it would also imply that GTST discordance has little effect on the ST topology. If the distance between a a test tree and Max.Discord.ST is low, that would imply that GTST discordance had a strong effect on the ST topology. To accomplish this, we calculated all pairwise RF and path distance, which were then added together, and rescaled between 0 and 1. We also calculated these as a single composite value called the “distance score” (S.1.3) where higher scores indicate a tree is more similar to the STs that have known low discordance (i.e., Concatenation.ST, Min.Discord.ST). Then, node-by-node topological comparisons were made to determine which specific relationships were attributable to maximal or minimal GTST discord.

The plausibility of the tested STs tells which GT sample yields the most realistic evolutionary scenario. Thus, each ST was evaluated under three criteria: internal consistency, predictive power, and congruence. High internal consistency was defined as high mean ASTRAL node support, high mean clade frequency in the underlying GTs, and mean concordance (RF distance) between the ST and underlying GTs. Predictive power was assessed by counting how often relationships in the ST were found in GTs (estimated under both models as described above) for 60 loci not used in the coalescent ST inferences. These were compared statistically by randomly resampling the 60 GTs with replacement for 100 trials. At each trial, the mean of the frequency of occurrences were taken each time and the distribution of means were compared via a Z-Test as implemented in the R package BSDA [3]. We also used a concatenation Approximately Unbiased Test framework (see Table S2.3.1 for the methodology and results). The Approximately Unbiased Test uses a distribution of RELL bootstrap trees to assess input tree plausibility given concatenated data [74]. Congruence was defined as presence of predefined control nodes (see S1.2), presence of previously hypothesized relationships, and presence of morphological support for relationships. See supplementary material S2.3 for justification and evidence for each. In comparison to the other criteria, this is more qualitative. Yet, it is important because it’s the most independent from our own study. Also, it considers expert knowledge, which is a critical aspect of interpreting empirical phylogenies.

In our final ST (SelAC.ST), we looked for candidate nodes in the anomaly zone in accordance with Linkem et al. [49]. We also identified the most difficult nodes as meeting four criteria: (i) having coalescent branch lengths < 0.1, (ii) gene concordance factor < 10%, (iii) split frequency among 100 GTs < 10%, (iv) ASTRAL node support < 0.5.

## Results

**Fig. 2:**
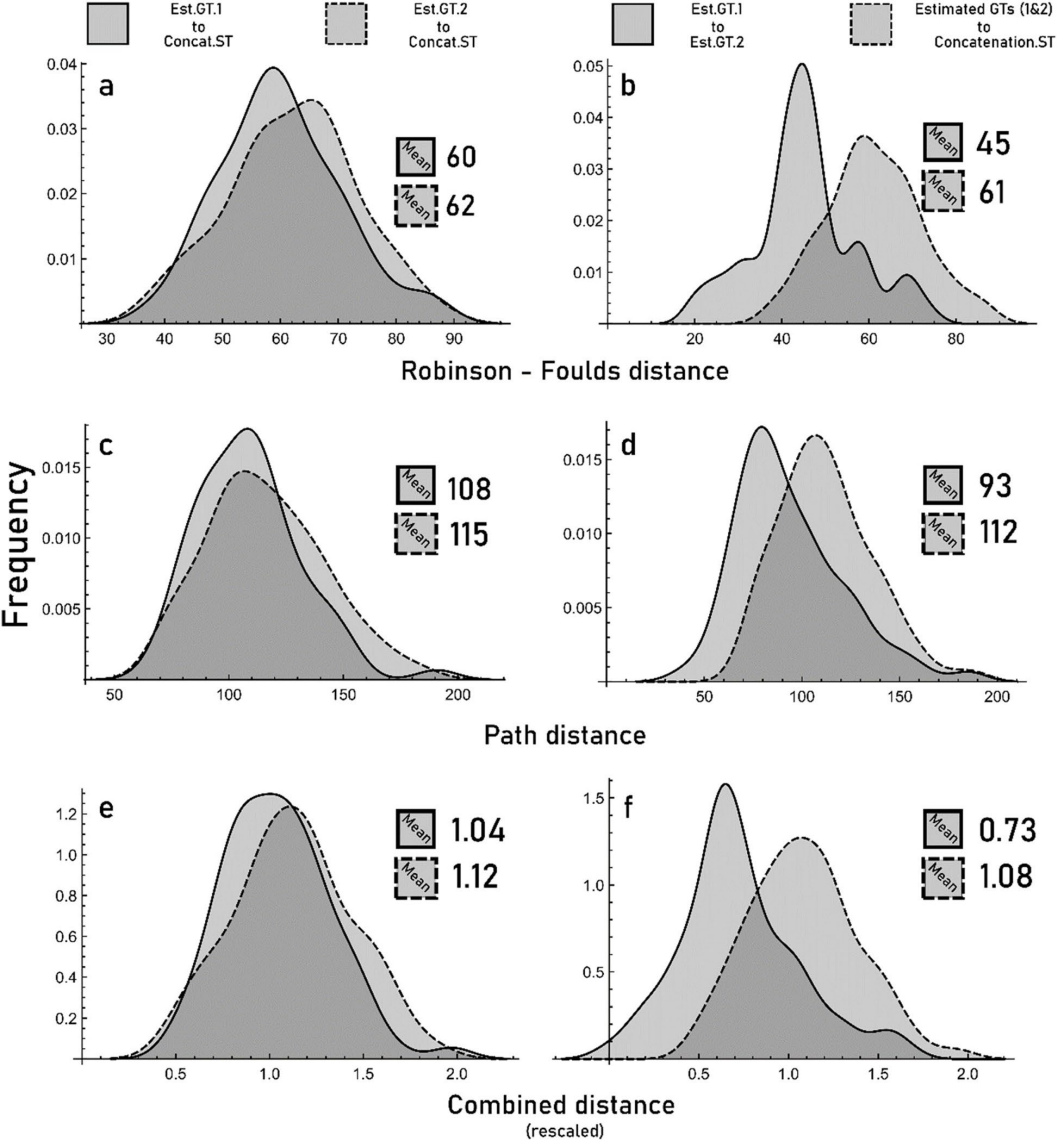
Histograms showing the distribution of topological distances among possible gene tree (GT) topologies. Robinson-Foulds distances are given in (**a**, **b**), path distance in (**c**, **d**) and a combined and rescaled distance is given in (**e**, **f**). The distances from the two estimated GTs (Est.GT.1 and Est.GT.2) to Concatenation.ST (all with equal taxon sampling) are shown in the left panels (**a**, **c**, **e**); the distance between both estimated GTs and the mean distance to Concatenation.ST are shown in the right panels (**b**, **d**, **f**). Means of each distribution in each panel are given to the right. Means, and their corresponding distribution, are labelled with the same line type (solid or dashed). Est.GT.1 tended to be slightly less discordant than Est.GT.2. Estimated GTs were more similar to each other than they were to the Concatenation.ST

The 40 alignments had a median parsimony score of 1076.5 (min. 260, max. 4711; based on the Est.GT.1 topologies). Gene tree-to-species tree (GTST) discordance was ubiquitous in every gene tree (GT) because no estimated GTs came within 38 RF (normalized RF = 0.47) of the Concatenation.ST (Fig. 2). This level of discordance is approximately equivalent to five random clade swaps in a 45-taxon tree. Each estimated topology was unique as they were never less than 20 RF (normalized RF = 0.23) distance from each other (Fig. 2). There were no splits that were recovered in 100% of GTs but one outgroup node (Xylophagodea) was recovered in > 90% of all GTs in which the constituent taxa were present (*n* = 30). The GTR model tended to estimate slightly less discordant GTs compared to the Empirical Codon Models (78% of cases; Fig. 2 a, c, e). With few exceptions, the GTs were more similar to each other than they were to Concatenation.ST (Fig. 2 b, d, f).

### Model tests on gene trees

**Fig. 3:**
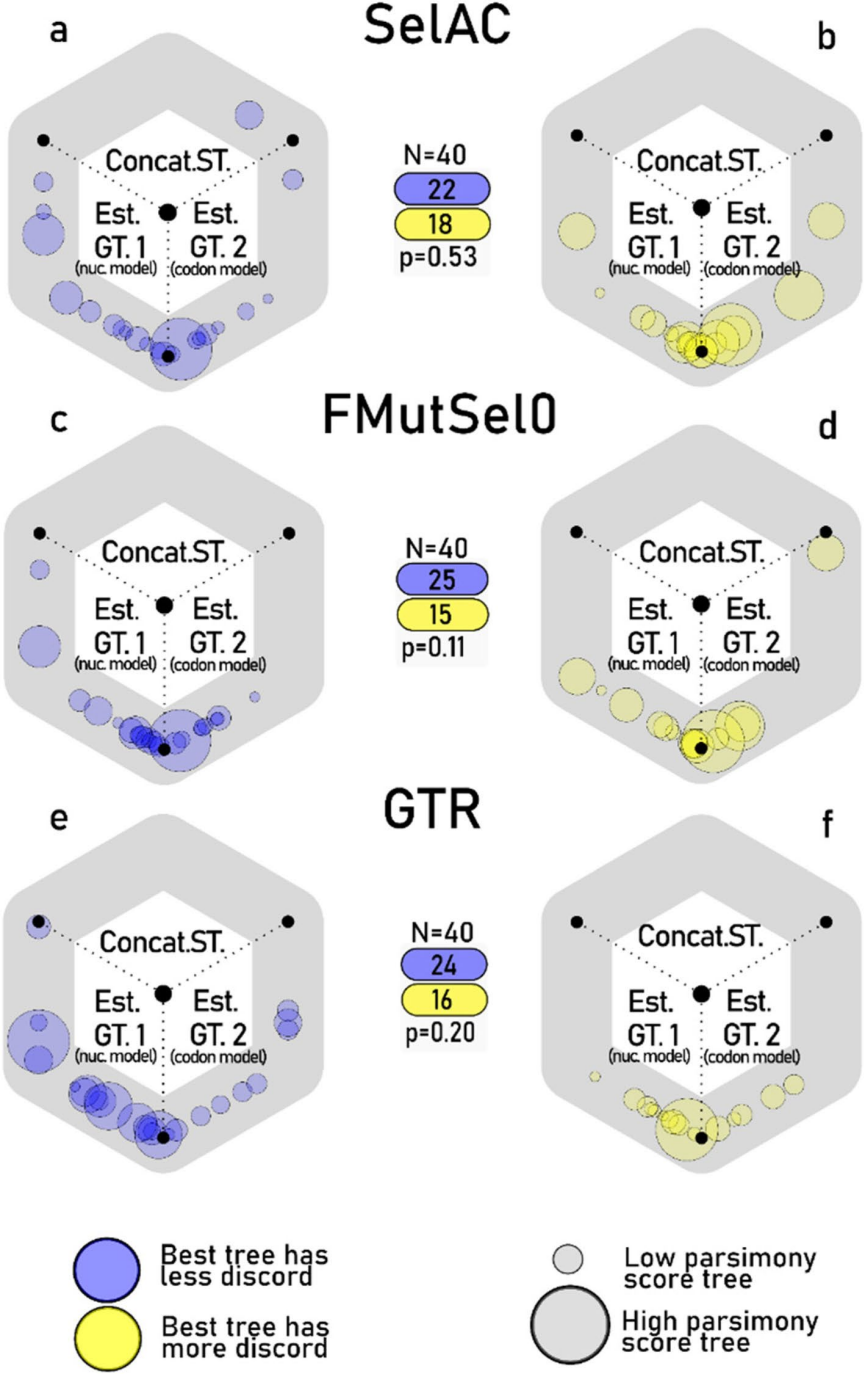
Results from the tests of 40 genes under three models: SelAC (**a**, **b**), FMutSel0 (**c**, **d**), and GTR (**e**, **f**). Circles along the hexagon each represent a genomic locus, its position represents the relative likelihood of the three possible tree topologies. For instance, a circle in the exact top (12 o’clock) position would represent a case where the Concat.ST topology for a locus was better than either alternative topologies for the same locus. Whereas a circle at the 11 o’clock position would show that the Concat.ST had the highest lnL, and the Est.GT.1 would have ranked second. In other words, the position of the circle around the hexagon is related to the ΔLnL. Circle radii indicate the parsimony scores of loci. The results are separated by cases where the models favored the trees with less (blue) or more (yellow) GTST discordance. For nearly all tests, the Concatenation.ST was the least favored, indicating that some GTST discordance was always preferred. However, there was no observed preference for more, or less GTST discordance relative to the Concatenation.ST. Most of the points are seen to cluster near the bottom, indicating that Est.GT.1 and Est.GT.2 were often very close in their lnL. This was particularly true with FMutSel0 and among the GTs with more discordance to the ST

**Fig. 4:**
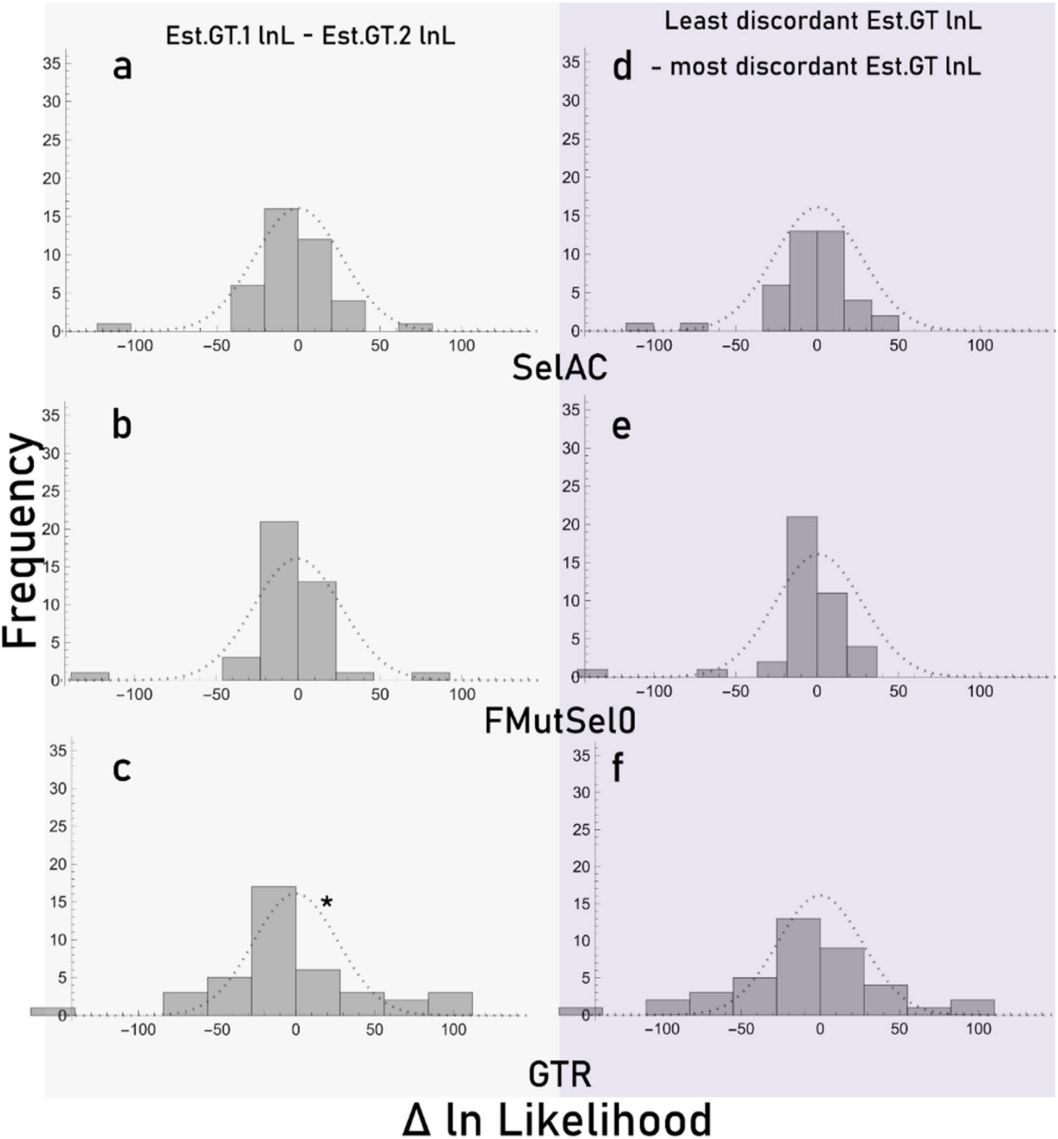
Histograms showing the distribution of delta ln-likelihoods (∆lnL) for the indicated GTs under the three models. ∆lnL between Est.GT.1 and Est.GT2 (**a**-**c**). ∆lnL between the Est.GT that was less discordant to Concatenation.ST and the Est.GT that was more discordant to Concatenation.ST (**d**-**f**). Dashed lines represent the null distribution expected as determined based on a randomized resampling of all the data. * indicates statistically significant deviation from the mean of the null expectation as determined from a Kolmogorov-Smirnoff test (α = 0.05). This test only considers the magnitude of preference for a certain estimated GT. The magnitudes of lnLs from GTR were slightly biased towards Est.GT.1

None of the models had a statistically identifiable preference for GTs with less GTST discordance (Fig. 3). The number of times the least discordant GT was identified as the maximum likelihood GT by the models are 22/40 (SelAC), 24/40 (GTR), and 25/40 (FMutSel0), which was not more than expected by randomness (*p* > 0.05). GTR (via SelAC package) preferred GTs estimated under GTR (via IQTREE; i.e., Est.GT.1) 65% of the time (*p* < 0.05). SelAC and FMutSel0 each preferred more Est.GT.1 topologies than Est.GT.2, but this preference was not statistically significant. The above results are from Z-Tests of mean comparisons, and we tried other tests using the same binary results (randomization test, sign tests) or ∆lnLs (Kolmogorov–Smirnov Test; Fig. 4). They all agreed.

**Fig. 5:**
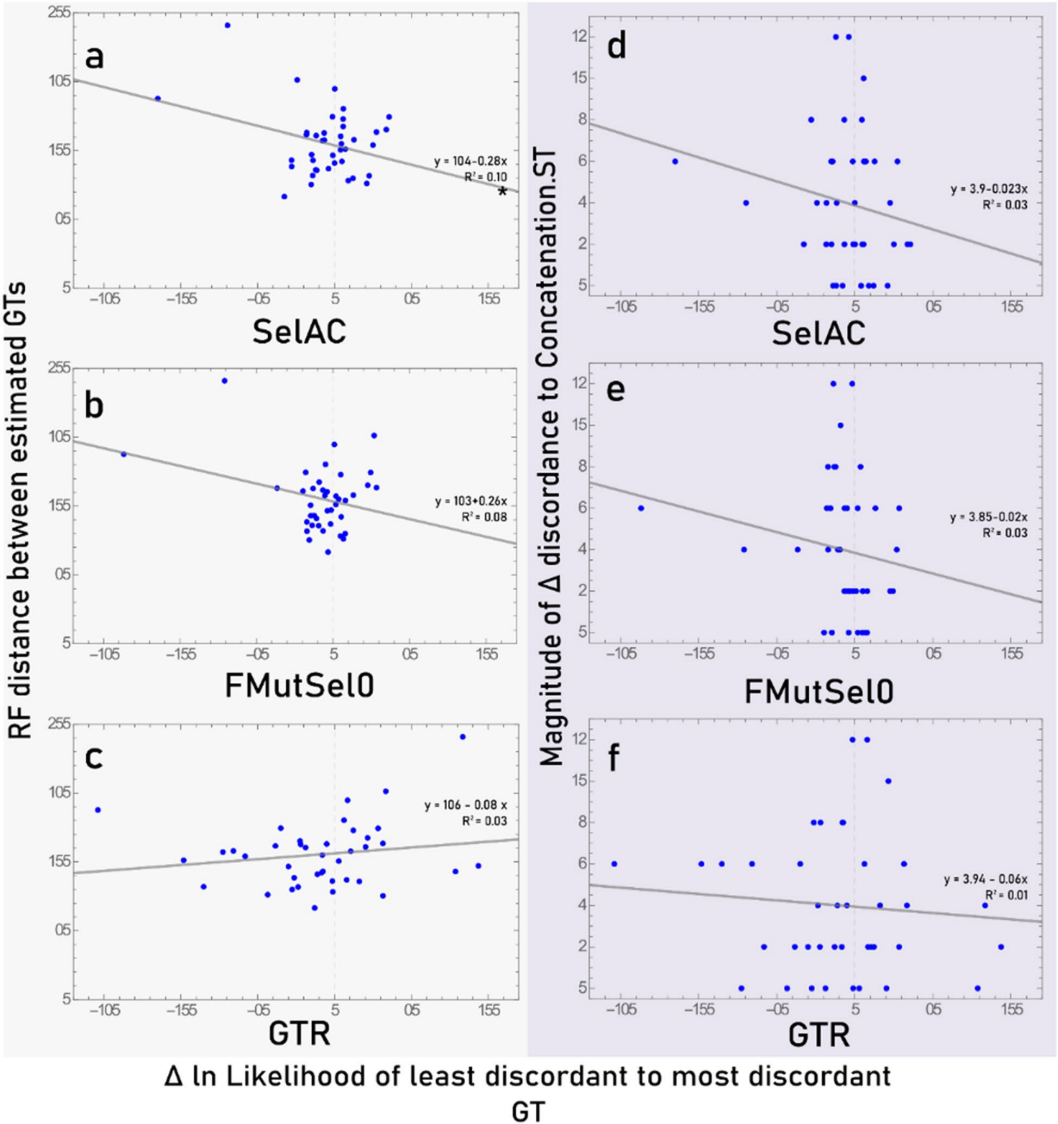
Relationship between preferences for the least discordant estimated gene tree (GT) and the amount of difference between the estimated GTs. Left panels show the differences between the two estimated GTs (**a**, **b**, **c**) and right panels show the magnitude of difference in discordance among the two estimated GTs (**d**, **e**, **f**). Data is fit with a linear model and statistical significance determined at an α level of 0.05. The only statistically significant regression (*) was found to be entirely driven by the presence the two outlying points on the left

Finally, there was no relationship between how distinct the estimated GTs were from each other (Fig. 5) and the lnL they received. The magnitude of discordance to the Concatenation.ST had no relationship to the magnitude of lnLs from any of the three models (Fig. 5 d-f). With SelAC, the difference in lnL did show a relationship to the topological distance between the two estimated topologies (Fig. 5 a). However, when the two outliers on the left are removed, the trend entirely disappears (*p* = 0.36, R^2^ = 0.02, m = 0.17).

The results were largely the same when repeating some of the procedure with reference to Naïve.Coal.ST. All RF distances from GTs to Naïve.Coal.ST were ≥ 40. Although, two of the 40 loci (Znev_00145, Znev_06187) would not have fulfilled our original criteria for inclusion in the experiment because RF and path distances did not agree on which GT (Est.GT.1 or Est.GT.2) was less discordant to the Naïve.Coal.ST. Also, one of the remaining 38 loci had a different least discordant GT (Znev_00897’s Est.GT.1 was less discordant to Naïve.Coal.ST while Est.GT.2 was less discordant to Concatenation.ST). Thus, this repeat analysis compared 38 loci, 20 of which had Est.GT.1 as the least discordant GT, and 18 of which had Est.GT.2 as the least discordant GT. Among this slightly reduced, and skewed, sample SelAC selected the least discordant GT 22 times (*p* = 0.33), FMutSel0 selected the least discordant GT 25 times (*p* = 0.04) and GTR selected the least discordant GT 22 times (*p* = 0.33). FMutSel0 did show a statistically significant preference for the less discordant GT, but this was a result of the slight change in the sample.

### Effects of GTST discordance on species trees

Details of the ST topologies, support for relationships under different scenarios, predictive ability of the STs, and detailed comparison of their overall plausibility are given in supplementary material S2.2 and S2.3. Figure S2.2.1 shows the differences in the topologies of the four test STs (Est.ST.1, Est.ST.2, SelAC.ST, FMutSel0.ST), a best-case scenario (Min.Discord.ST), worst case scenario (Max.Discord.ST) and baseline tree (Concatenation.ST).

Although the 40 GT test set was fairly divided among low discordance GTR trees (Est.GT.1) and low discordance codon model trees (Est.GT.2), Est.ST.1 was the least similar to Max.Discord.ST, and moderately similar to Concatenation.ST. FMutSel0.ST showed the most similarity to Min.Discord.ST and Concatenation.ST. Est.ST.1 had the best distance score of 0.4, FMutSel0.ST and SelAC.ST had distance scores of 0.36 and 0.37 and Est.ST.2 had the poorest distance score (0.28; Table 1, S2.2.1). Yet, as mentioned previously, none of the GT sets underlying any of these STs was significantly skewed towards being more or less discordant with the Concatenation.ST.

**Table 1:** Comparison of species trees (STs)

Species Tree (ST) Name	Data	Criteria for choosing Gene Trees (GT)	Gene tree model	Analysis	Distance score^a^	Selected relationships score^b^
Concatenation.ST	265 loci	No discordance to ST^d^	NA	RAxML (partitioned, GTR)	0.68	19
Min.Discord.ST	40 least discordant GTs	Minimal discordance to ST^d^	GTR or ECMS05^**c**^	ASTRAL	0.64	12
Max.Discord.ST	40 most discordant GTs	Maximal discordance to ST^d^	GTR or ECMS05^**c**^	ASTRAL	0.09	−8
Est.ST.1	40 Est.GT.1 topologies	Model based lnL	GTR	ASTRAL	0.40	0
Est.ST.2	40 Est.GT.2 topologies	Model based lnL	ECMS05	ASTRAL	0.28	−3
SelAC.ST	40 SelAC chosen GTs	Model based lnL	GTR or ECMS05^**c**^	ASTRAL	0.36	−9
FMutSel0.ST	40 FMutSel0 chosen GTs	Model based lnL	GTR or ECMS05^**c**^	ASTRAL	0.37	4

Selected relationships in the FMutSel0.ST are most consistent with low discordance relationships when measured against the concatenation topology. Est.ST.2 and SelAC.ST are the least consistent

^a^Distance score is a composite of scaled Robinson-Foulds (RF) and path distances from the discordance-based STs (Concatenation.ST, Min.Discord.ST, Max.Discord.ST). The value is higher when distance from the Concatenation.ST and Min.Discord.ST is lower and distance to the Max.Discord.ST is higher

^b^ Selected relationship score is higher when a tree shares a certain relationship (see supplemental data) with the concatenation and Min.Discord.ST and is lower when it shares a relationship with the max. discordance tree

^c^Topologies inferred from the GT models were subsequently chosen based on the better of the lnL scores from a second set of models (GTR, SelAC, FMutSel0). ECMS05 = Empirical Codon Model [71]

^d^^”^ST” refers to the Concatenation.ST

We used three criteria to determine the plausibility of the test STs: internal consistency, power to predict independent GT topologies, and congruence with previous studies. Est.ST.1 had the highest mean local posterior probability, and the FmutSel0 and SelAC.STs were the most consistent with the GTs in its underlying set (considering mean split frequencies and RF distances; Table 2). As a second test of internal consistency, we used an Approximately Unbiased Test to compare likelihoods of each ST topology given a concatenated alignment. We tested this with an alignment of 265 loci and 40 loci both without partitioning. We did a final test with the 265 loci alignment that used gene and codon position partitioning (Table S2.3.1). Concatenation.ST was deemed plausible under all alignments, and the only coalescent topology not rejected (α = 0.05) was SelAC.ST under the partitioned 265 loci alignment (∆lnL = 829.84, *p* = 0.59).

**Table 2:** Metrics to evaluate the plausibility of each species tree (ST)

		**Species Tree (ST)**						
	**N**	**Concatenation**	**Min. discord**	**Max. discord**	**Est.ST.1**	**Est.ST.2**	**SelAC.ST**	**FMutSel0.ST**
Mean ASTRAL support^a^	40	NA	76%	76%	79%	76%	77%	75%
Mean split freq. of GTs the ST was calculated from^b^	40	35%^6^	37%	35.30%	36.81%	35.51%	37.42%	37.93%
Mean RF distance among GTs the ST was calculated from^b^	40		56.85	59.45	58.15	58.35	57.20	57.50
Frequency of relationships occurring in independent set of GTs^c^	120	NA	30%	30%	28.8%* ± 2.0%	29.1% ± 2.2%	29.2% ± 1.9%	29.4% ± 2.3%
Control nodes present^d^	9	9	7	7	6	7	7	7
Congruence evidence for^e^	11	NA	-	-	7	1	2	2
Congruence evidence against^f^	11	NA	-	-	−8	−6	−6	−6

The values under each ST indicate its plausibility under the criteria of: internal consistency with underlying data, predictive ability, and congruence with independent data

^a^ Mean of all node support values (local posterior probability of quadripartition)

^b^ Internal consistency of the data set expressed as the mean of all bipartition frequencies occurring in the sampled gene trees (GTs) and mean Robinson-Foulds (RF) distance among the sample GTs

^c^ A measure of the ST’s ability to predict independent GT topologies. Values are provided with 95% confidence intervals (from bootstrapping). Asterisks denote that the values are significantly less than one (*) or more (**) of the others

^d^ The number of predefined control nodes that are found in the ST

^e^ Number of relationships that have independent evidence (morphological or published phylogenetic) arguing for or against their veracity

^f^ This value is the mean bootstrap support of the Concatenation.ST (from RAxML)

The STs that predicted the highest number of relationships found in an independent sample of 120 GTs (from 60 loci) were: FMutSel0 (29.4% ± 2.3%), SelAC (29.2% ± 1.9%), Est.ST.2 (29.1% ± 2.2%), and Est.ST.1 (28.8% ± 2.0%) (Table 2). These were all deemed statistically insignificant differences, except for Est.ST.1, which had less predictive power than the others (α = 0.05).

As one test of congruence, we looked for a set of control nodes − relationships known with high certainty (see supplementary material S1.2). Only the Concatenation.ST had all nine control nodes present. Each test ST had six (Est.ST.1) or seven (Est.ST.2, SelAC, FMutSel0) control nodes present − differing with each other only in which lineage was sister to Blattellidae.

As a second test of congruence, we evaluated which relationships in the test STs had prior support from published morphological studies or independent phylogenetic analyses. Of the 11 Blaberidae taxa whose sister relationships we examined (Fig. S2.3.3), the relationships in Est.ST.1 were deemed most plausible (Table 2; see supplementary material S2.3 for specific information) and SelAC and FMutSel0 were tied for second. In Est.ST.1 there were six relationships with putative independent support and six relationships with independent data conflicting with them. In FMutSel0.ST and SelAC.ST, there were two relationships with supporting data and four relationships with conflicting data. The Est.ST.2 tree had one relationship supported and four relationships conflicted.

## Discussion

When inferring a set of GTs among an increasing number of genes and taxa, there will always eventually be GTST discordance at one node at least. With our modest sample size of Blaberidae and outgroups (56 taxa, 40 genes), we found that all estimated GTs had GTST discordance at multiple nodes (Fig. 2). This is expected given the clade age (~ 60 Ma; [26]) and taxonomic breadth of the data [68]. Yet, even though GTST discordance was ubiquitous in all GTs, the amount of discordance (e.g., the number of GT nodes discordant with the ST) can still be low or high. We assessed the amount of discordance in two ways: in an experimental framework, and through comparisons of STs. The hypothesis tested in our experiment, that more complex evolutionary models will prefer GT topologies with less discordance as opposed to ones with more discordance, was not supported by the results (Fig. 3). The extent of GTST discordance had no relationship with model optimized lnL (Fig. 5) in our controlled set of 40 genes. We then compared the robustness of coalescent STs inferred with multiple combinations of GT sets (outlined in Fig. 1). The aims were to compare different criteria of choosing GTs to analyze in ASTRAL and determine which resulted in a better phylogeny and were thus most realistic. Est.ST.1, utilizing only GTR optimized GTs, showed topological similarities to STs that assumed low GTST discordance (Table 1). Yet, Est.ST.1 had the lowest power to predict independent GT topologies (frequency of relationships occurring in an independent set of GT = 28%), and it was missing one well-established control node in the outgroup. Est.ST.2 was the least similar to STs assuming low GTST discordance and was also the least internally consistent. FMutSel0.ST and SelAC.ST had better metrics than the previous STs. They both had moderate similarity to the ST topologies that minimized GTST discordance (Table 1) and had similar internal consistency, predictive ability, and congruence with previous studies (Table 2. The Approximately Unbiased Test was the only framework where we see a meaningful advantage of the SelAC.ST over FMutSel0.ST (Table S2.3.1, making it, by a slim margin, the best inferred ST. Thus, coalescent inference of the SelAC chosen GTs yield a more robust ST, but it was not the best ST under all criteria and was significantly lacking in congruence with previous studies (compared to Est.ST.1; but note that the vast majority of previous studies did not use a coalescent method. It also came with a substantial computational cost relative to other methods, though that is likely due to its implementation rather than anything intrinsic in this class of models. Regardless, STs inferred from gene sets with higher GTST discordance were easily ruled out, and STs with low/moderate levels of discordance all had similarly strong quality metrics.

We cannot make straightforward conclusions about the nature of GTST discordance, minimizing GT error, or the value of complex codon models for GT inference. Simulation studies demonstrate that minimizing GTST discordance results in less accurate STs [94], and our analyses may support that as well. We do see that model choice, GT topologies, and the amount of discordance have profound effects on coalescent ST topologies (Fig. S2.2.1) that are not evident in tree-wide support metrics (e.g., mean node support, or split frequencies between ST and GTs). Similar observations have been made in other studies [6].

The use of complex codon models (FMutSel0 and SelAC) did seem to improve the ST, if only slightly (Fig. S2.2.1; Table 2). In particular, the GT set selected by SelAC resulted in a coalescent ST that reduced internal GTST discordance. Other studies have seen that their most robust STs also reduce or minimize GTST discordance [6, 76]. While the experimental framework did not find that less GTST discordance was preferred, this was perhaps due to calculation of GTST discordance relative to naïve STs (Concatenation.ST and Naïve.Coal.ST). Still, given the extreme computational cost of optimizing these models on single genes (see Methods section), the theoretical arguments against some complex models like the MutSel family of models [5, 38, 41, 58, 77, 92], and the demonstrated strong performance of using only GTR (Tables 1, and 2), we are not wholly convinced of the benefit to using more complex models alone to minimize GT error. That being said, our experimental framework only tested a narrow subset of all substitution models and did not vary other factors at all. For instance, a more robust approach would involve testing the fit of all substitution (e.g., [40]), rate heterogeneity [85], branch-length linkage [19], and heterotachy [13] models. It may be that more complex codon models would have shown greater improvements for some GTs when these other aspects of the overall evolutionary model were optimized for each gene.

Our results suggest that systematic error, and probably also estimation error, are indeed abundant in estimated GTs and that complex models may reduce them. While we cannot know with certainty which GT topologies are closer to the “truth”, the simpler models (GTR, Empirical Codon Model) always disagreed on the GT topology (100% of cases; *n* = 100), but the complex models (SelAC, FMutSel0) disagreed with each other in only 10% of cases (*n* = 40). Any instances of disagreement are cases where there are certainly errors in at least one GT. Since individual genes may not have enough character information to inform accurate GTs [66], GT systematic errors doubly compound this problem. This emphasizes the importance of utilizing longer contigs [88], leveraging approaches that co-estimate GTs and STs (e.g., [28, 34, 63, 90]), or using multi-locus information to inform quartets on the ST under the coalescent model [10]. Indeed, errors have a meaningful effect on the downstream ST if they are unaccounted for (Table 1, Fig. S2.2.1, S2.3.3). Of course, joint estimation of STs and GTs is also vulnerable to errors from ineffective modelling of alignments [64]. Given this, studies whose results hinge largely on the prevalence of incomplete lineage sorting may need to be examined more closely [12].

### Limitations

There are some other caveats to highlight. First, our original hypothesis favored SelAC because it was previously found to outperform others (including FMutSel0; [5]). Yet this was not evident in our analysis. In addition to the limitations discussed above, the way we utilized SelAC here did not take full advantage of its functionality. SelAC is designed to estimate parameters from whole multi-locus datasets [5], so limiting its optimization to single genes is slower, and potentially less accurate. Next, loci with minimal rate heterogeneity were chosen to minimize modelling issues [9, 95], although other factors should also be considered when selecting optimal loci [17, 23, 55, 57, 79]. Minimizing among site rate heterogeneity could be disadvantageous if it resulted in higher rate heterogeneity among lineages [98]. Though we found no difference (Z-test, *p* > 0.05) in the performance of either FMutSel0 or SelAC in identifying the least discordant GT among the 14 least and 14 most heterogeneous loci (Table S2.1.2). A related issue, minimizing among site rate heterogeneity could decrease our ability to see the improvements that selection-based codon models would have over GTR. A larger limitation of the study is the lack of a simulation framework to test our experimental question. Finally, GT errors are often due to long branch effects [66, 87], which is not explicitly what was tested in this study. Given the difficulty in objectively identifying long branch effects in any tree (e.g., [24, 43], let alone a set of GTs, we do not attempt at quantifying it.

There are also some key limitations to the ST inference and choice. First, due to the presence of anomalous nodes, the ST may not reliably predict GT split frequencies [14]. So, this test is limited in its ability to identify the most robust ST. Along the same lines, additional improvements to ST choice could be made by incorporating more nuanced elements of GT support [6, 68, 75]. In the ST inference itself, a potential limitation is that, by utilizing the SelAC chosen GTs (or FMutSel0 GTs) in a coalescent analysis, we may be violating a core assumption of the multi-species coalescent model – that GTs are drawn from purely random evolutionary processes [14]; although the GTs themselves were inferred using GTR and the Empirical Codon Model). Also, as mentioned earlier, our simplifying assumptions about rate heterogeneity [40, 85], and heterotachy [13] are likely to be biologically implausible. Other assumptions of the multi-species coalescent are also probably violated (e.g., that GTs are all drawn from the same distribution, [19]). Since we have shown that the ST is hugely sensitive to the distribution of GTs (because of limited number of loci and many anomalous nodes), we expect that many of these are non-trivial issues. In short, there is certainly more work to be done on the Blaberidae phylogeny. With this in mind, we can consider what we may have learned from the present analysis.

### Implications for Blaberidae

**Fig. 6:**
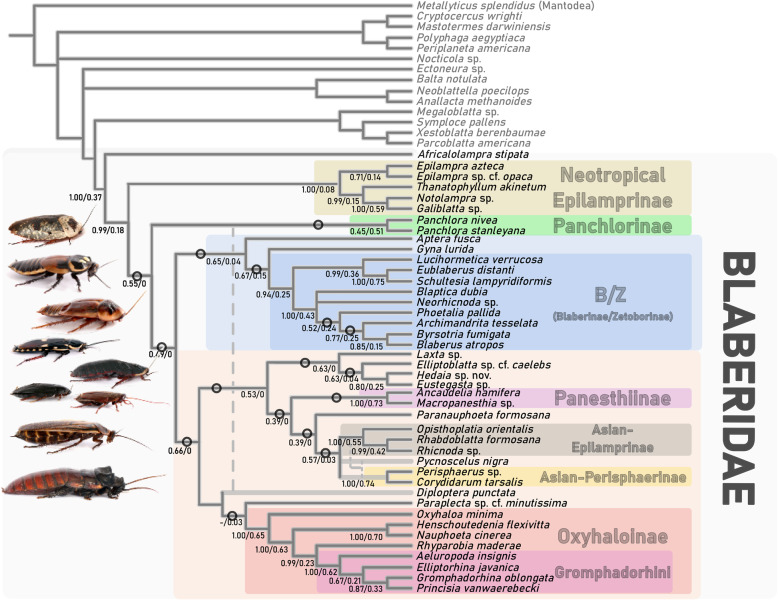
Species tree of Blaberidae. Inferred from an ASTRAL III analysis of GTs of 40 loci as chosen by the SelAC model with modifications (dashed grey) based on tests conducted herein. Values on edges are local posterior probability for the quartet (ASTRAL)/frequency of the split (bipartition) in 120 gene trees inferred from 60 independent genes under two models (GTR, Empirical Codon Model). Support values only shown for ingroup. Circles indicate anomalous nodes

These findings have some novel implications for the phylogeny of Blaberidae, but interpreting them requires some context. Evangelista et al. [23] inferred a phylogeny of Blaberoidea, including 48 species of Blaberidae, with a concatenated analysis of 264 genomic loci. That study went to great lengths to infer a robust ST by identifying the most informative genomic loci. However, being a concatenation analysis, it might be expected to poorly recover anomalous nodes [54]. That study and other recent ones [21, 22, 50, 91] suggest that many nodes within Blaberidae may be functionally unresolvable. We identified 16 internal nodes as fulfilling the anomaly zone criteria in Linkem et al. [49], Fig. 6). An analysis of another dataset utilizing 1183 GTs totaling > 1 million nucleotide positions [21] did not refute this, and also identified 16 internal nodes in Blaberidae as being in the anomaly zone, including many of the same nodes identified here (although the topologies and taxon sampling are different; Fig. 6).

Some relationships were consistently recovered in all coalescent analyses but were not found with concatenation − suggesting that GTST discordance needs to be accounted for on these nodes. This was the case in finding *A. fusca* as sister to *Gyna lurida* + B/Z (Blaberinae/Zetoborinae; ASTRAL node support = 0.65); and *Princisia vanwaerebecki* sister to *Gromphadorhina oblongonota* (ASTRAL node support = 0.87). The latter is a relationship suspected by morphological similarity (pers. comm. George Beccaloni), as opposed to the relationship proposed by concatenation [23].

The SelAC.ST (Fig. 6), which was the most plausible ST, has Neotropical Epilamprinae as sister to the remaining Blaberidae except *Africalolampra stipata* (ASTRAL node support = 0.99). This is congruent with the concatenation topology [23] and another recent study [22] but not other phylogenomic studies [7, 21]. Similarly, the successive placement of Neotropical Epilamprinae and Panchlorinae as pectinate with the remaining Blaberidae conflicts with most other studies [7, 23, 24, 48, 91].

One of the most common GT topologies (29.3%) was Panchlorinae as sister to *D. punctata.* This was recovered in previous studies (reviewed in [24]) but may be a long-branch artifact. When placed together, both lineages have long unbranched tips on a short stem. Given the moderate, but enticing, morphological and molecular (Est.ST.1 and Min.Discord.ST) support for *D. punctata* in a clade with *Paraplecta* and Oxyhaloinae (S2.3) we have grafted Fig. 6 to reflect both relationships. Three other studies [7, 21, 50] place Oxyhaloinae deeply in Blaberidae and one [91] places it deeply in Blaberidae and sister to Diplopterinae. Evangelista, et al. [21] even demonstrated that signal favored a deep placement of Oxyhaloinae over alternatives and recovered this as the best ST in a coalescent analysis. Thus, we have different lines of evidence suggesting that Diplopterinae, *Paraplecta* and Oxyhaloinae form a clade, and that this clade may be sister to all other Blaberidae except the clade containing *A. stipata.* Future studies should target these taxa, and explicitly try to test support for these relationships to perhaps settle this issue.

Evangelista, et al. [26], with small taxon sampling, showed that the major lineages of extant Blaberidae (*Panchlora*, *Gyna lurida*, B/Z, *D. punctata*, Oxyhaloinae) originated within a span of ~ 35 Myr. after the origin of crown-Blaberidae. Our ST (Fig. 6) suggests two reasons for potentially revising this perception of Blaberidae’s diversification towards and even more rapid series of speciation events. First, we show that between four and eight additional diversification events occurred along the backbone of Blaberidae during this period (diversification of Peri-Indian Blaberidae; the splitting of *Paraplecta minutissima* and *Oxyhaloa minima* from other Oxyhaloinae; splitting of neotropical-Epilamprinae and *A. fusca* from their respective sister groups). This could drive a node density effect [37, 86] and increase the time since divergence and unpredictably change the duration of the clade’s diversification. Second, Evangelista, et al. [26] did not include *A. stipata* or any other African-Epilamprinae like *Africalolampra* [61], which recent studies [22, 23, 91] and the present one find to be sister to the remaining Blaberidae with strong support (ASTRAL node support = 1). Including these lineages may have shifted the location of the fossil calibration relative to other Blaberidae lineages and thus may have narrowed the timeframe of diversification. A novel divergence date analysis is needed to clarify these issues.

## Conclusions

Our original prediction was that most gene tree-to-species tree discordance was a combination of systematic and estimation error, and thus more realistic evolutionary models would minimize gene tree discordance with the RAxML concatenation species tree or a naïve coalescent species tree. This was not supported by our main experiment. Taken at face value, one might conclude that gene tree-to-species tree discordance is not low and therefore it should be accounted for by using multi-species coalescent models. Yet, choosing gene tree topologies with more realistic evolutionary models (SelAC and FMutSel0) did yield a species tree that was, on average, less discordant with gene trees. In this case, the one might conclude that decreasing gene tree discordance does improve species tree topologies but that the multi-species coalescent model is still necessary because the concatenation topology could not recover this result. Of course, these conclusions would differ in the presence of abundant introgression, yet the need for using coalescent models would be even greater.

We also found that gene tree-to-species tree discordance was never absent and that the complex models we tested (SelAC and FMutSel0) appear to strongly reduce gene tree error. The species tree inferred from gene trees chosen by the SelAC model were determined to be the best under three criteria but almost as good as those chosen by the FMutSel0 model and even gene tree topologies from GTR only were more strongly supported in some metrics. Regardless, the model used to choose gene tree topologies had large effects on the coalescent species tree, and they all had important differences from the concatenation topology. We identify eight deep lineages specifically affected by seemingly small differences among the gene tree set, and 16 Blaberidae nodes as being within an anomaly zone.

## Supplementary Information

Supplementary Material 1.

## Data Availability

The datasets supporting the conclusions of this article are available in the DRYAD repository at 10.5061/dryad.0cfxpnw8n.
