# Supplementary material for: Concatenation fails to describe the anomalous radiation of giant cockroaches (Blattodea: Blaberidae) despite moderate to low discordance
Source: BMC Ecol Evol. 2025 Jul 21;25:72. doi: 10.1186/s12862-025-02409-4 (PMC12278584; doi:10.1186/s12862-025-02409-4)
Supplement: Supplementary file 1 — Supplementary Material 1. [file 12862_2025_2409_MOESM1_ESM.docx]

Supplement to

Concatenation fails to describe the anomalous radiation of giant cockroaches (Blattodea: Blaberidae) despite evidence of generally low discordance

Dominic A. Evangelista, Michael Gilchrist, Frederic Legendre, Brian O’Meara


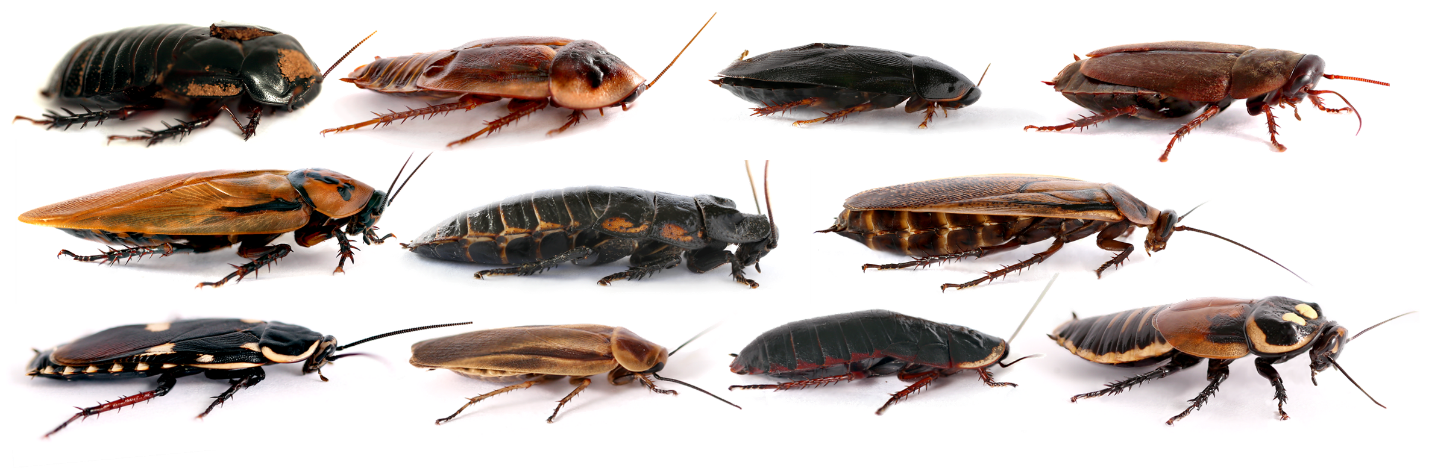


# S.1 Supplementary methods

## S.1.1 Choice of evolutionary models and model search parameters

The goal of inferring gene trees under different models is to examine cases where there are multiple plausible topologies for a given locus and come to conclusions about differing discordance with the species tree. For this comparison to be biologically meaningful both alternatives must be robust hypotheses given different assumptions. We chose the first hypothesis to be dictated by the assumptions of the GTR model. GTR is an extremely widely used nucleotide model, as evidenced by the popularity of phylogenetic software that only implements this model (Stamatakis 2014). It is the most complex and versatile of nucleotide models (Tavaré and Miura 1986) and thus should yield meaningful evolutionary histories in most cases. Additionally, GTR is the only nucleotide model that could be implemented in both our tree inference software (IQ-TREE) and our tree testing software (SelAC). We decided the second hypothesis should also be meaningful but should incorporate codon-level information, since codon models are thought to be superior to nucleotide models (e.g., Arenas 2015; Doud, et al. 2015; Goldman and Yang 1994; Sealfon, et al. 2015; Wang, et al. 2014). However, there is little consensus on which codon model would be most appropriate. In order to ensure that the codon model was meaningful, we allowed some flexibility. Before each inference of the second hypothesis IQ-TREE’s built in model-finder determined if ECMS05 (Schneider, et al. 2005) or ECMK07 (Kosiol, et al. 2007) and which frequency model was most appropriate. These models differ in that: GTR only considers nucleotide changes and estimates substitution frequencies from the data (Tavaré and Miura 1986), both ECM models consider codon structure, ECMS05 uses observed substitution rates from a large vertebrate dataset (Schneider, et al. 2005), and ECMK07 uses physicochemical properties of amino-acids to estimate codon evolutionary rates (Kosiol, et al. 2007). ECMK07 also allows for instantaneous doublet or triplet mutations to occur (Kosiol, et al. 2007). Surprisingly, ECMS05 was chosen as superior for all loci.

The goal of testing alternative gene tree topologies with SelAC, and FMutSel0 were to use the most biologically plausibly models, despite their complexity. The incredible complexity of protein evolution justifies such approaches considering that many parameters varying across individual sites. For instance, evolutionary rate of any given amino acid is known to vary, increasing if it is: on the protein surface, contributing to protein flexibility, in a region of protein structural disorder, in a lowly expressed protein, not necessary to maintain a stable structure, or not playing a role in the active region of the protein (Echave, et al. 2016). Determining evolutionary dynamics based on these mechanistic predictors requires biophysical models (Echave, et al. 2016), which SelAC does not explicitly take into consideration but can approximate through heterogeneity of the strength of selection. Related to variable rate evolution of amino acid sites, there is also evolutionary preference for certain amino-acid states at specific sites (Doud, et al. 2015; Risso, et al. 2015; Wang, et al. 2014). It has also been hypothesized that sites with changing amino-acid preferences positionally correlate with sites that are fast evolving but this has not always been demonstrated (Doud, et al. 2015). Both SelAC and FMutSel0 consider site specific amino-acid preferences, which tend to strongly outperform models lacking such parameters (Doud, et al. 2015). Finally, both rate of evolution and the site-specific preferences for certain amino-acids have epistatic effects (Hoehn, et al. 2017) and these features may or may not change over time (Risso, et al. 2015; Usmanova, et al. 2015). Again, SelAC does not model these explicitly, but provides a framework under which the resulting patterns can be modelled.

### Table S1.1.1

Search parameters used in gene tree optimization runs impliemented in the SelAC R package.

| Independent starts in run | Chain length | Search iterations per run |
| --- | --- | --- |
| 1 | 1000 | 6 |
| 3 | 1300 | 4 |
| 3 | 1300 | 4 |
| 5 | 2000 | 6 |

## S.1.2 Control nodes and justification

One method by which we can assess the plausibility for a species tree is to judge whether it contains nodes, or relationships that have been well-established with strong support (i.e., Shen, et al. 2017). In other words, an estimated species tree should reliably recover uncontroversial relationships. Below are eight relationships among the taxa we included in our analyses that we deem uncontroversial. The prior evidence to support this is discussed.

1. **Monophyletic Solumblattodea.** The relationships among the three super-families of Blattodea have often been in flux (e.g., Djernæs, et al. 2015; Grandcolas 1996; Inward, et al. 2007; Klass and Meier 2006; Legendre, et al. 2015; Liu, et al. 2023; McKittrick 1964; Murienne 2009). However, Evangelista, et al. (2019a) recovered the Solumblattodea hypothesis with a phylotranscriptomic dataset with strong support via multiple tests for data bias, and outlined morphological support as well. Thus, we expect that this clade should appear in a strong species-tree.
2. **Monophyletic Pseudophyllodromiidae sensu (Evangelista, et al. 2020)**. A controversy regarding the placement of *Anallacta* with respect to Blattellinae and Pseudophyllodromiinae was discussed in Evangelista, et al. (2019a), who placed the genus as sister to Pseudophyllodromiinae. This overturned the previous placement in Blattellinae (Grandcolas 1996), which was also shown to be unsupported in Bourguignon, et al. (2018). Evangelista, et al. (2019b) and Evangelista, et al. (2020) showed high support for *Anallacta* as sister to Pseudophyllodromiinae with multiple tests. Evangelista, et al. (2020) included more taxa and was thus able to discern morphological characters supporting the monophyly of *Anallacta* + *Lobopteromorpha*. Thus, we expect that this clade should appear in a strong species-tree.
3. **Monophyletic Blattellidae + Nyctiboridae.** Klass and Meier (2006) and Grandcolas (1996) gave multiple character-state changes supporting the sister relationship between Blattellinae and Nyctiborinae. Recent phylogenomic studies have also recovered this clade, and with strong support (Evangelista, et al. 2020; Evangelista, et al. 2019b; Liu, et al. 2023). Thus, we expect that this clade should appear in a strong species-tree.
4. **Monophyletic Orkrasomeria**. Klass and Meier (2006) give seven morphological character-state changes supporting the monophyly of Blattellinae + Nyctiborinae + Blaberidae (= Orkrasomeria). Recent studies (Evangelista, et al. 2020; Liu, et al. 2023; Wang, et al. 2023) recover it with strong support. Thus, we expect that this clade should appear in a strong species-tree.
5. **Monophyletic Neotropical-Epilamprinae**. The monophyly of world-wide Epilamprinae is not supported by molecular evidence (Bourguignon, et al. 2018; Evangelista, et al. 2018; Evangelista, et al. 2019b; Legendre, et al. 2017; Legendre, et al. 2015). Morphological studies treating Neotropical genera of Epilamprinae (Roth 1970a, c, 1971b) show evidence for the monophyly of some Neotropical tribes (Epilamprini, Poeciloderrhiini, Notolamprini and Colapteroblattini) but do not give explicit evidence for their monophyly. Recent molecular studies sampled Epilamprini and Colapteroblattini and have supported their monophyly (Bourguignon, et al. 2018; Legendre, et al. 2017) along with the genus *Thanatophyllum*, which we consider to be a Neotropical Epilamprinae. Thus, we expect that this clade should appear in a strong species-trees.
6. **Monophyletic Blaberinae/Zetoborinae (B/Z)**. Grandcolas (1993) recovered this group as monophyletic (and the two constituent subfamilies each monophyletic) with three supporting character state changes. This agreed with the precladistic opinion by McKittrick (1964). Later, molecule based or combined-data studies (Djernæs, et al. 2012; Evangelista, et al. 2020; Evangelista, et al. 2018; Legendre, et al. 2017; Liu, et al. 2023) similarly demonstrate monophyly of a group containing both subfamilies (although the constituents are usually paraphyletic with respect to one another, and not containing *Thanatophyllum*). Thus, we expect that this clade should appear in a strong species-tree.
7. **Monophyletic Oxyhaloinae**. Roth (1971a) discussed morphological evidence for the monophyly of this clade. Subsequent studies corroborated this (Bourguignon, et al. 2018; Evangelista, et al. 2020; Evangelista, et al. 2018; Evangelista, et al. 2019b; Legendre, et al. 2017; Liu, et al. 2023; Wang, et al. 2023). Thus, we expect that this clade should appear in a strong species-tree.
8. **Monophyletic Gromphadorhini**. This clade is strongly supported by some recent molecule-based studies (Bourguignon, et al. 2018; Evangelista, et al. 2020; Inward, et al. 2007). One recent study didn’t support its monophyly with respect to the genus *Leozehntera* (Legendre, et al. 2017). However, the representatives of Gromphadorhini we sample here are monophyletic in all of these studies. There are also numerous synapomorphic morphological (sexually-dimorphic pronotal morphology, wings absent, strong sclerotization, huge body size) and behavioral (e.g., hissing) characters supporting their monophyly. Thus, we expect that this clade should appear in a strong species-tree.

## S.1.3 Distance score

A composite “distance score” was calculated to categorize the STs as being more consistent with maximal or minimal GTST discordance. Distance score was calculated as $\left( \frac{\left( D_{max}-D_{concat} \right)}{D_{max}}+\frac{\left( D_{max}-D_{minDisc} \right)}{D_{max}}+\frac{\left( D_{maxDisc} \right)}{D_{max}} \right)/3$ where D_max_ is the maximum distance between any ST pair, D_concat_ is the distance to Concatenation.ST, D_minDisc_ is the distance to the Min.Discord.ST, and D_maxDisc_ is the distance to the Max.Discord.ST. The distances used were RF and path distances which were then added together. High distance scores indicate that a tree is more consistent with a set of GTs that are minimally discordant with the ST. Then topological comparisons were made to determine which recovered relationships were attributable to maximal or minimal GTST discord.

## S.1.4 Terminology of gene trees and species trees

### Table S.1.4.1.

We used different terminology for various gene trees and species trees at different points in our study. While we have attempted to standardize these in the main text, and figures, data files, code and this supplementary text may contain other terminology. This table clarifies these. This table may be missing some alternate usages or spellings, but the list is comprehensive enough for the reader to understand.

| Term used in main text | Type | Terms possibly used elsewhere | Description |
| --- | --- | --- | --- |
| Concatenation.ST | Species tree from RAXML  or an approximation of a possible gene tree | concatTree, Concat. species tree, Concatenation species tree, Concat.ST, RAXML tree, Concatenation topology, concat.ASTRAL | A phylogenetic species tree topology inferred by concatenating all data and then variously pruned to fit specific analyses. |
| Est.GT.1 | Gene tree from IQTree2 | GTRGTree, estimated gene tree 1, estimated topology 1, GTR gene tree, GTRGMedian.tre | Any phylogenetic gene tree topology inferred under the GTR+G4+FO model with the median used to estimate rate categories (as opposed to the mean). |
| Est.GT.2 | Gene tree from IQTree2 | CODONTree, estimated gene tree 2, estimated topology 2, ECM gene tree, CODON.tre | Any phylogenetic gene tree topology inferred under the ECMS05+G4 model with a nucleotide frequency model determined by ModelFinder. |
| Est.ST.1 | Species tree from ASTRALIII | Estimated species tree 1, GTR ASTRAL tree, estTop1GTR.ASTRAL | A single phylogenetic species tree inferred by finding the best multi-species-coalescent tree of 40 Est.GT.1s |
| Est.ST.2 | Species tree from ASTRALIII | Estimated species tree 2, ECM ASTRAL tree, estTop2CODON.ASTRAL | A single phylogenetic species tree inferred by finding the best multi-species-coalescent tree of 40 Est.GT.2s |
| SelAC.ST | Species tree from ASTRALIII | SelAC species tree, SelAC ASTRAL tree | A single phylogenetic species tree inferred by finding the best multi-species-coalescent tree of 40 gene trees ranked best by SelAC |
| FMutSel0.ST | Species tree from ASTRALIII | FMutSel0 species tree, FMutSel0 ASTRAL tree | A single phylogenetic species tree inferred by finding the best multi-species-coalescent tree of 40 gene trees ranked best by FMutSel0 |
| Min.Discord.ST | Species tree from ASTRALIII | Minimum discordance tree, Congruence ASTRAL tree | A single phylogenetic species tree inferred by finding the best multi-species-coalescent tree of 40 gene trees among 80 total Est.GT.1 Est.GT2. The criteria used to choose the GT for each locus was minimizing GTST discordance with Concatenation.ST. |
| Max.Discord.ST | Species tree from ASTRALIII | Maximum discordance tree, Incongruence ASTRAL tree | A single phylogenetic species tree inferred by finding the best multi-species-coalescent tree of 40 gene trees among 80 total Est.GT.1 Est.GT2. The criteria used to choose the GT for each locus was maximizing GTST discordance with Concatenation.ST. |

# S.2 Supplemental results

## S2.1 Model tests: 66 loci

We did preliminary tree optimizations for 66 loci. They had on average, 45 taxa (min. 34, max. 51) per locus. Branch lengths for each of the three trees for each locus were optimized again using the SelAC software package (Beaulieu, et al. 2019). We fit: GTR+G4+FO, SelAC+GTR+G4+FO+amino acid optimization (AAO), and MutSel+GTR+G4+FO+AAO. In both cases, estimated branch lengths from IQ-TREE were used as starting values and optimization chain was run for a single set of initial conditions with six iterations of 1000 evaluations each and the criteria max.tol.edges = 1.4 and tol.step = 2.3, parallelized over two processors.­­­­­ The log-likelihood (lnL) was used to determine which gene tree was preferred by which model.

The results from testing all 66 loci with three models in the SelAC software package are as follows. The simplest evolutionary model (GTR) assigned the highest lnLikelihood (lnL) to estimated topology 1 70% of the time. The SelAC and FMutSel0 models also assigned the highest lnL to estimated topology 1 a majority of the time (59-64% of the time). Each model assigned the highest lnL to a less discordant topology more often than to a more discordant topology but only FMutSel0 did so in a manner that was statistically significant compared to random tree choice (i.e., p<0.05). Despite the overall trend towards reducing gene-tree discordance, the species-tree topology was rejected in an overwhelming majority of tests. The statistical significance of model family on a topology’s optimality under the three models was more apparent. GTR was much more likely (p<0.05) to choose estimated topology 1 than estimated topology 2 (presumably because estimated topology 1 was inferred with GTR). FMutSel0 (P<0.05) was less likely to choose estimated topology 2 (estimated with another codon model). SelAC was indistinguishable from randomness when comparing against either model family.

### ***Table S2.1.1***

The best trees chosen for all loci tested. Statistical significance is denoted * and was determined using a Z-Test with alpha = 0.05. FMutSel0 significantly chose less discordant gene-trees more often than more discordant ones. However, FMutSel0 and GTR both showed a significant bias for trees generated under a certain model family (i.e., they preferred estimated topology 1 over estimated topology 2).

### ***Table S2.1.2***

Comparison of gene tree optimizations of loci with different levels of among site rate heterogeneity within 66 loci. Rate heterogeneity is calculated by determining the mean number of rate categories per nucleotide [specifics of calculation in (Evangelista, et al. 2020)]. The values in the table show how often the models correctly identified the least discordant tree. P-values show the probability that the least discordant tree was identified at the same rate in both heterogeneity sets as determined by a Z-Test.

|  | **% of hits on the least discordant gene tree** | |  |
| --- | --- | --- | --- |
|  | **Low Heterogeneity** | **High Heterogeneity** | ***p*** |
| **SelAC** | 43% | 53% | 0.87 |
| **FMutSel0** | 48% | 57% | 0.28 |
| **GTR** | 48% | 38% | 0.09 |
| *n* | *14* | *14* |  |

## S2.2 Comparison of six species trees

### ***Figure S2.2.1***


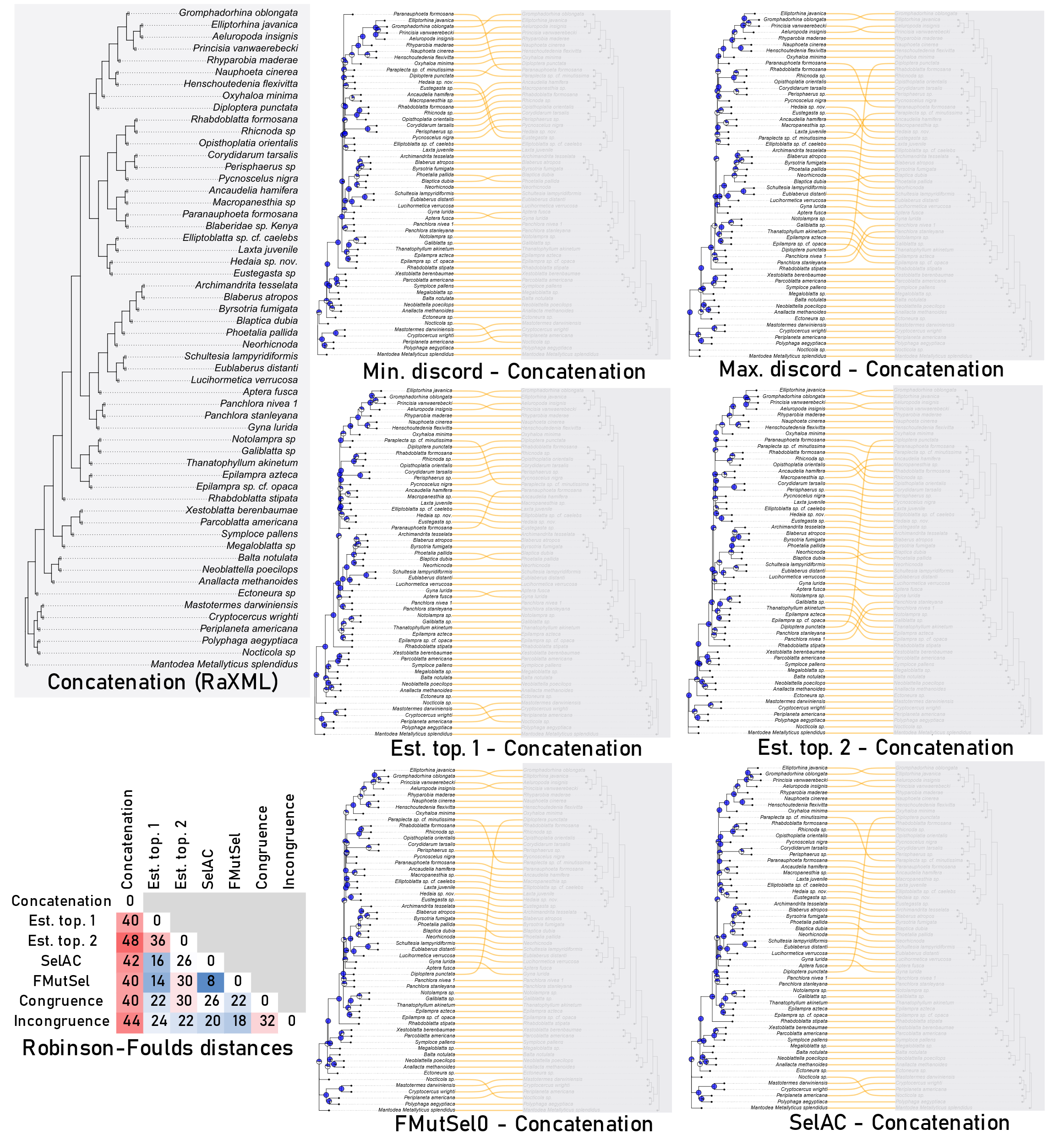
The full concatenation tree topology compared to six species-trees inferred in this study. (a) Concatenation.ST inferred in (Evangelista, et al. 2020) with taxa trimmed. (b-g) “Cophylo” plots of six species-trees against the Concatenation.ST shown in (a). (b,c) Species-trees inferred from all the most discordant 40 gene-trees and the most discordant 40 gene-trees. (d, e) Species-trees inferred from all 40 gene-trees of Est.ST.1 and Est.ST.2 respectively. (f, g) Species-trees inferred from all 40 gene-trees selected by FMutSel0 and SelAC respectively. Blue pies on nodes show local posterior probability support. (h) Symmetrical matrix of Robinson-Foulds (RF) distances among all species trees. Colors emphasize high (red) and low (blue) RF distances.

### ***Table S2.2.1***

Pairwise, and combined distance scores^1^ for all species trees.

|  | **Concatenation.ST** | **Est.ST. 1** | **Est.ST. 2** | **SelAC.ST** | **FMutSel.ST** | **Min.Discord.ST** | **Min.Discord.ST** | **Distance score^2^** |
| --- | --- | --- | --- | --- | --- | --- | --- | --- |
| **Concatenation.ST** | 0.00 |  |  |  |  |  |  | 0.68 |
| **Est. ST. 1** | 0.83 | 0.00 |  |  |  |  |  | 0.40 |
| **Est. ST. 2** | 0.96 | 0.79 | 0.00 |  |  |  |  | 0.28 |
| **SelAC.ST** | 0.82 | 0.51 | 0.72 | 0.00 |  |  |  | 0.36 |
| **FMutSel.ST** | 0.76 | 0.43 | 0.70 | 0.29 | 0.00 |  |  | 0.37 |
| **Min.Discord.ST** | 0.92 | 0.66 | 0.73 | 0.71 | 0.65 | 0.00 |  | 0.64 |
| **Max.Discord.ST** | 0.90 | 0.66 | 0.55 | 0.59 | 0.51 | 0.78 | 0.00 | 0.09 |

^1^ Each pairwise value is the sum of the pairwise Robinson-Foulds (RF) distance divided by the overall maximum RF distance and the pairwise Path distance divided by the overall maximum path distance. Lower values indicate less topological distance.

^2^ This composite distance score is the sum of three distance scores as described in S1.3.

## S2.3 Morphological and other support for recovered relationships

One relationship recovered in both Concatenation.ST and most of the coalescent trees (Est.ST.1 FMutSel0.ST, Min.Discord.ST, and Max.Discord.ST) was Pycnoscelinae as sister to Asian Perisphaeriinae. While the local posterior probability of the relationship is low (FmutSel0.ST 0.36), this relationship appears in independent gene trees at a high rate (10.8%) and is also congruent with the Concatenation.ST. The Est.ST.2 and SelAC.ST had different relationships. Roth (1973b) showed morphological evidence for a relationship between Pycnoscelinae, Diplopterinae and Oxyhaloinae (but see McKittrick 1964) but we never found this topology. A relationship with Asian Perisphaerinae or Diplopterinae both make sense biogeographically − all are distributed in South-East Asia. There has previously been no consistent molecular (Bourguignon, et al. 2018; Djernæs, et al. 2012; Evangelista, et al. 2018; Legendre, et al. 2014; Legendre, et al. 2017; Legendre, et al. 2015) hypothesis for Pycnoscelinae.

We did consistently recover the clade Asian-Epilamprinae + *Pycnoscelus* + Asian-Perisphaerinae across all coalescent species trees and this was also recovered in Liu, et al. (2023). This, and other relationships recovered only by ASTRAL (Figs. S2.2.1, S2.3.3), are prime suspects for relationships affected by ILS. The major differences between the coalescent species-tree and Concatenation.ST are in four locations.

First, the deep relationships among “Peri-Atlantic” Blaberidae. All the coalescent trees agreed that Gyninae (only *Gyna* *lurida* was sampled) was sister to the Blaberidae/Zetoborinae (BZ) complex and *Aptera fusca* was sister to both, and Panchlorinae was sister to the remaining Peri-Atlantic Blaberidae. In contrast, our Concatenation.ST, and that of a recent phylotranscriptomic studies (Evangelista, et al. 2019b; Liu, et al. 2023), showed Gyninae as more closely related to Panchlorinae [although neither study sampled *Aptera fusca*]. Little morphological systematic work has been done to clarify the positions of these taxa (but see Grandcolas 1993). Both topologies offer equally parsimonious biogeographical scenarios (two transitions each) but depend on the timing of the splits in coordination with the drift of S. America away from Africa. However, Legendre, et al. (2017) and unpublished data (FL) indicate that more taxa may be needed to precisely clarify this region of the tree (e.g., *Gynopeltis* spp., other putative Gyninae, and African Perisphaerinae).

Second, ASTRAL-III always recovered *Blaberus atropos* as sister to *Byrsotria fumigata* instead of sister to *Archimandrita tessellata,* which is the concatenation hypothesis. Roth (1970b) indicates that *Blaberus* and *Archimandrita* are most closely related (also see Legendre, et al. 2017). Thus, the ASTRAL hypotheses are at odds with the previous hypotheses for these genera.

Third is the position of *Phoetalia pallida* and *Blaptica dubia*, which was sister to *Byrsotria fumigata + Archimandrita tessellata + Blaberus atropos* in all the coalescent trees while it was sister to *Blaptica dubia* + *Byrsotria fumigata* + *Blaberus atropos* + *Archimandrita tessellata* in Concatenation.ST. The position of *Phoetalia* was previously debated by McKittrick (1964), Roth (1970b), Legendre, et al. (2017) and Wang, et al. (2023) but the debate was largely concerning its relationship to the “Blaberinae” complex or the “Zetoborinae” and not its position relative to *Byrsotria*.

Finally, *Gromphadorhina oblongata* was sister to *Princisia vanwaerebecki* in all ASTRAL trees but sister to *Aeluropoda insignis + Elliptorhina javanica + Princisia vanwaerebecki* in the Concatenation.ST. *Princisia* and *Gromphadorhina* are very morphologically similar (two robust pronotal horns and a raised anterior pronotal margin) and thus are thought to be closely related [pers. obs. Evangelista; G. Beccaloni pers. comm.; also see (Legendre, et al. 2017)]. The putative synapomorphies uniting *Princisia* and *Gromphadorhina* could be symplesiomorhies though. Particularly considering the earlier branching of *Leozehntnera* (Legendre, et al. 2017), which has a similar pronotal morphology. Genital morphology is relatively conserved in this group, and thought to be uninformative.

Relationships recovered in the positive control (Min.Discord.ST) coalescent tree but not in the negative control tree (Max.Discord.ST) are those that align with our hypothesis that most gene tree discordance is due to gene tree error and not ILS (i.e., the least discordant gene tree is probably the correct gene tree). Of the nine times where this was true (among 18 examined relationships), only one occurred in the test trees. Est.ST.1, FMutSel0.ST, and SelAC.ST had *Paraplecta minutissima* as sister to Oxyhaloinae. *Paraplecta* was suggested to be more closely related to Perisphaeriinae based on the excavation of the right side of the subgenital plate [(Roth 1995); also see (Roth 1973a)]. However, the excavation is hooked like in Oxyhaloinae (who have hooks on both the left and right sides). Est.ST.1 tree and the Min.Discord.ST (positive control) species tree also have Diplopterinae (*Diploptera punctata*) as sister to *Paraplecta minutissima +* Oxyhaloinae. The alternative (seen in the Est.ST.2, FMutSel0.ST, and SelAC.ST) is Diplopterinae as sister to Panchlorinae. This alternative was also found in some other molecule-based studies (Evangelista, et al. 2018; Legendre, et al. 2017) but this may be due to long-branch attraction. A close relationship between Diplopterinae and Oxyhaloinae was a prevailing morphological hypothesis in precladistic morphological studies (reviewed in Li and Wang 2015) and was also found in recent phylogenomic studies (Bourguignon, et al. 2018; Evangelista, et al. 2020; Evangelista, et al. 2019b; Wang, et al. 2023). If true, and since we always recovered *Oxyhaloa duesta* as sister to the remaining Oxyhaloinae, it would suggest that a “coleopteriod” body form (dorso-ventrally thickened, strongly tegmenized or elytriform forewings, greatest body width more than half medial body length; seen in *Diploptera punctata*, *Paraplecta minutissima*, and *Oxyhaloa duesta*) is the plesiomorphic state to Oxyhaloinae and a the more traditionally roachoid formed (dorso-ventrally flattened; moderately or lightly tegmenized forewings; greatest body width less than half medial body length) is autapomorphic in the clade.

Relationships recovered in the negative control (Max.Discord.ST) coalescent topology and not the positive control (Min.Discord.ST) topology are those that disagree with our hypothesis. If true, they would indicate that most gene-tree discordance with the species-tree are due to ILS. We see two instances of this among the test species trees. First, Max.Discord.ST and Est.ST.1 trees with Panesthiinae and *Laxta* sp. and second between the Max.Discord.ST, and Est.ST.2 trees with respect to Paranauphoetinae and Oxyhaloinae. In Max.Discord.ST and Est.ST.1, Panesthiinae and *Laxta* sp. are sister taxa. This is at odds with the monophyly of Perisphaerinae, which has a morphological basis (Anisyutkin 2003; Grandcolas 1997). Yet, it could be the case that *Laxta* sp. is correctly positioned as sister to Panesthiinae and the other Perisphaerinae are the ones misplaced (Anisyutkin 2003). *Laxta* has previously been considered as an Epilamprinae by both morphological (Roth 1992) and molecular (Bourguignon, et al. 2018) determination. The Max.Discord.ST, and Est.ST.2 trees both recovered Paranauphoetinae as sister to Oxyhaloinae. There is no known morphological support for this. Anisyutkin (2003) proposed that Paranauphoetinae was sister to Perisphaerinae + Panesthiinae with three morphological character states supporting the monophyly of all three taxa and three morphological characters separating Paranauphoetinae from the other two.

We also see that Concatenation.ST, FMutSel0.ST, and Max.Discord.ST topologies support Asian-Epilamprinae as sister to Pycnoscelinae + Asian-Perisphaerinae. This relationship could be found in all trees due to a common source of error (i.e., signal erosion) in the individual gene alignments and concatenated alignments. We discuss above that Pycnoscelinae as sister to Asian-Perisphaerinae was recovered in most trees. However, the only known phenotypic support for this relationship is their shared biogeographical range. The same is true for Asian-Epilamprinae − the taxa we included all reside in S.E. Asia. Given that Epilamprinae (sensu Roth 2003) are prominently distributed in S. America and Africa one might think that the recovered topology represents a conflict with the morphological hypothesis for the group. However, it is already suspected that Epilamprinae is polyphyletic (Bourguignon, et al. 2018; Evangelista, et al. 2020; Evangelista, et al. 2023; Legendre, et al. 2017; Liu, et al. 2023; Wang, et al. 2023). The lineages in question were included in two prior molecule-based study support was low in both and the relationships have few analogs here (Legendre, et al. 2017; Wang, et al. 2023). Thus, we consider prior studies to be agnostic towards any specific relationship of Asian-Epilamprinae.

### ***Table S2.3.1***

Plausibility of species trees as determined by approximately unbiased (AU) tests and node support. Each AU test was done with the GTR+G model and a different concatenated dataset: 265 loci without partitioning, 265 loci with codon positions in optimized partitions, and 40 loci with no partitioning. The difference from the best likelihood (∆lnL), and the p-value (p) is given for each tree in each test. Trees with p<0.005 are considered implausible given the alignment. Plausible test results are marked with *. ASTRAL inferred tree from SelAC gene trees was plausible given a concatenated alignment of 265 loci with partitioning, but all other alignments and trees tested were deemed implausible.

|  |  | **AU Test Results** | | | | | |  |
| --- | --- | --- | --- | --- | --- | --- | --- | --- |
|  |  | **265 loci, no partitioning** | | **265 loci, codon partitioning** | | **40 loci, no partitioning** | | |
| **Species Tree** | **Type** | ∆lnL | p | ∆lnL | p | ∆lnL | p | |
| Concatenation.ST | baseline (RaXML) | 0.00 | 1.00* | 0.00 | 1.00 | 0.00 | 0.99 | |
| Min.Discord.ST | + control (ASTRAL) | 818.28 | 0.00 | 833.58 | 0.00 | 166.29 | 0.00 | |
| Max.Discord.ST | - control (ASTRAL) | 798.65 | 0.00 | 803.62 | 0.00 | 209.72 | 0.00 | |
| Est.ST.1 | ASTRAL | 705.93 | 0.00 | 722.85 | 0.00 | 162.70 | 0.00 | |
| Est.ST.2 | ASTRAL | 1646.66 | 0.00 | 1660.09 | 0.00 | 318.76 | 0.00 | |
| SelAC.ST | ASTRAL | 818.52 | 0.00 | 829.84 | 0.59* | 92.51 | 0.00 | |
| FMutSel0.ST | ASTRAL | 813.69 | 0.00 | 823.74 | 0.00 | 173.97 | 0.00 | |

### ***Table S2.3.2***

Frequency of relationships found in 120 independent gene trees. Percentages give the frequency that the defined relationships were recovered in a data set of gene trees inferred from 60 loci not included in the species tree inference. Gene trees were inferred in IQTREE with the two methods described in the main text. Cells are colored by percentage so high percentages are red and low percentages are blue. NAs indicate the relevant taxon combination was not present in any gene trees.

### ***Figure S2.3.3***


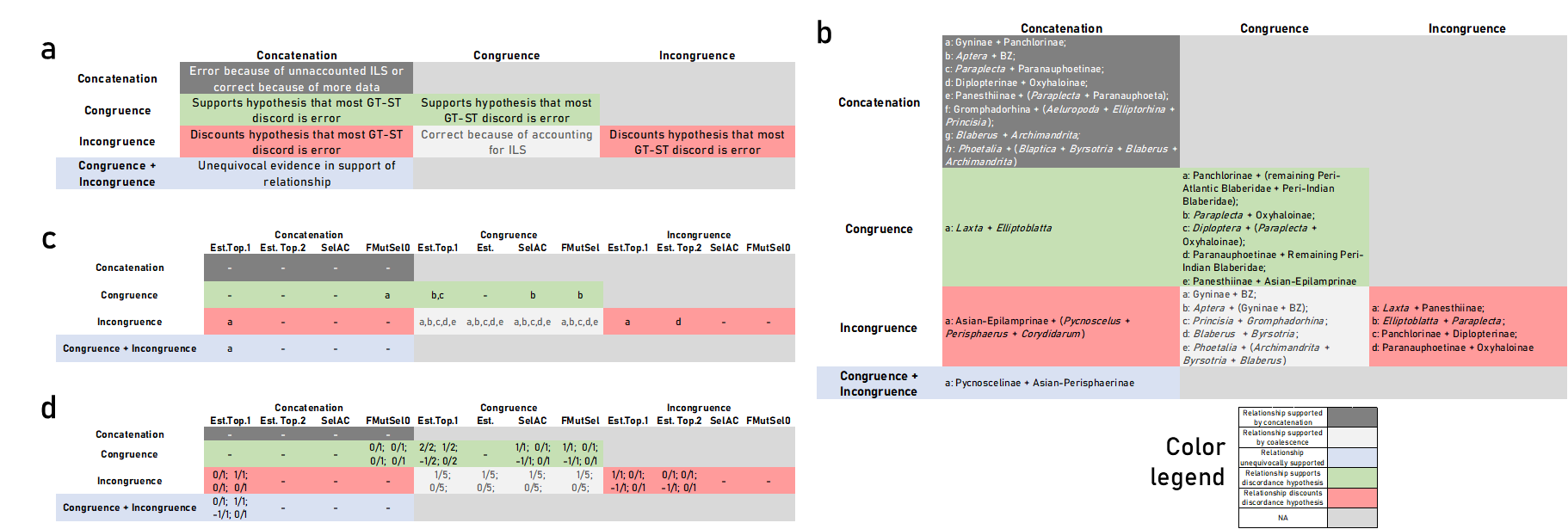
Comparison of unique relationships found in each species tree. Each table is organized to differentiate which relationships correspond to a gene-tree population that is highly congruent or highly incongruent, and which result from concatenation or coalescent inference. Each table is organized so that the cells discuss relationships uniquely supported by (from top left to bottom right) only Concatenation.ST, the Concatenation.ST and Min.Discord.ST (referred to as congruence), only Min.Discord.ST tree, the Concatenation.ST and Max.Discord.ST (referred to as incongruence) etc. (a) Explanation of how a relationship could be interpreted. (b) Unique relationships found in the baseline (Concatenation.ST) and control trees. (c) The relationships found in test trees. Letters correspond to those in panel (b). (d) Independent evidence for support of each relationship. Each fraction is the number of relationships supported (positive) or discounted (negative) by independent evidence (numerator) and the total number of relationships in that category (denominator). The values separated by semi-colons correspond to: (i) Strong morphological support for relationship; (ii) other support for relationship; (iii) strong morphological support for alternative; (iv) other support for alternative. "Other support" includes support in Evangelista, et al. (2019b) or other phylogenetic studies (Bourguignon, et al. 2018; Evangelista, et al. 2018; Legendre, et al. 2017; Liu, et al. 2023; Wang, et al. 2023); but not (Evangelista, et al. 2020). Support for most of the relationships is discussed in section S2.3 above.

# Works Cited

Anisyutkin LN 2003. On the systematic position of the subfamilies Perisphaeriinae Brunner, Panesthiinae Brunner, and the genus *Paranauphoeta* Brunner (Blattina: Blaberidae). Proc. Zool. Inst. Russ. Acad. Sci. 299: 25-32.

Arenas M 2015. Trends in substitution models of molecular evolution. Front. Genet. 6: 319. doi: 10.3389/fgene.2015.00319

Beaulieu JM, et al. 2019. Population Genetics Based Phylogenetics Under Stabilizing Selection for an Optimal Amino Acid Sequence: A Nested Modeling Approach. Molecular Biology and Evolution 36: 834-851. doi: 10.1093/molbev/msy222

Bourguignon T, et al. 2018. Transoceanic dispersal and plate tectonics shaped global cockroach distributions: Evidence from mitochondrial phylogenomics. Molecular Biology and Evolution 35: 1-14. doi: doi:10.1093/molbev/msy013

Djernæs M, Klass K-D, Picker MD, Damgaard J 2012. Phylogeny of cockroaches (Insecta, Dictyoptera, Blattodea), with placement of aberrant taxa and exploration of out-group sampling. Systematic Entomology 37: 65-83. doi: 10.1111/j.1365-3113.2011.00598.x

Djernæs M, Klass KD, Eggleton P 2015. Identifying possible sister groups of Cryptocercidae+Isoptera: A combined molecular and morphological phylogeny of Dictyoptera. Molecular Phylogenetics and Evolution 84: 284-303. doi: 10.1016/j.ympev.2014.08.019

Doud MB, Ashenberg O, Bloom JD 2015. Site-Specific Amino Acid Preferences Are Mostly Conserved in Two Closely Related Protein Homologs. Molecular Biology and Evolution 32: 2944-2960. doi: 10.1093/molbev/msv167

Echave J, Spielman SJ, Wilke CO 2016. Causes of evolutionary rate variation among protein sites. Nat. Rev. Genet. 17: 109-121. doi: 10.1038/nrg.2015.18

Evangelista D, et al. 2020. Assessing support for Blaberoidea phylogeny suggests optimal locus quality. Systematic Entomology 46: 157-171. doi: 10.1111/syen.12454

Evangelista D, Thouzé F, Kohli MK, Lopez P, Legendre F 2018. Topological support and data quality can only be assessed through multiple tests in reviewing Blattodea phylogeny. Molecular Phylogenetics and Evolution 128: 112-122. doi: 10.1016/j.ympev.2018.05.007

Evangelista DA, Nelson D, Varadínová ZK, Legendre F 2023. Phylogenomics and deep convergence in cockroach hind-wing morphology. Organisms Diversity & Evolution. doi: 10.1007/s13127-023-00609-8

Evangelista DA, et al. 2019a. An integrative phylogenomic approach illuminates the evolutionary history of cockroaches and termites (Blattodea). Proceedings of the Royal Society B: Biological Sciences 286: 1-9. doi: 10.1098/rspb.2018.2076

Evangelista DA, et al. 2019b. An integrative phylogenomic approach illuminates the evolutionary history of cockroaches and termites (Blattodea). Proceedings of the Royal Society B: Biological Sciences 286: 1-9. doi: 10.1098/rspb.2018.2076

Goldman N, Yang Z 1994. A Codon-based Model of Nucleotide Substitution for Protein-coding DNA Sequences. Molecular Biology and Evolution 11: 725-736.

Grandcolas P 1993. Monophylie et structure Phylogenetique des [Blaberinae+Zetoborinae+Gyninae+Diplopterinae] (Dictyoptera:Blaberidae). Ann. Soc. Entomol. Fr. 29: 195-222.

Grandcolas P 1997. The monophyly of the subfamily Perisphaeriinae (Dictyoptera: Blattaria: Blaberidae). Systematic Entomology 22: 123-130.

Grandcolas P 1996. The phylogeny of cockroach families: A cladistic appraisal of morpho-anatomical data. Canadian Journal of Zoology 74: 508-527.

Hoehn KB, Lunter G, Pybus OG 2017. A Phylogenetic Codon Substitution Model for Antibody Lineages. Genetics 206: 417-427. doi: 10.1534/genetics.116.196303

Inward D, Beccaloni G, Eggleton P 2007. Death of an order: A comprehensive molecular phylogenetic study confirms that termites are eusocial cockroaches. Biology Letters 3: 331-335. doi: 10.1098/rsbl.2007.0102

Klass K-D, Meier R 2006. A phylogenetic analysis of Dictyoptera (Insecta) based on morphological characters. Entomologische Abhandlungen 63: 3-50.

Kosiol C, Holmes I, Goldman N 2007. An empirical codon model for protein sequence evolution. Molecular Biology and Evolution 24: 1464-1479. doi: 10.1093/molbev/msm064

Legendre F, et al. 2014. The evolution of social behaviour in Blaberid cockroaches with diverse habitats and social systems: Phylogenetic analysis of behavioural sequences. Biological Journal of the Linnean Society 111: 58–77.

Legendre F, Grandcolas P, Thouzé F 2017. Molecular phylogeny of Blaberidae (Dictyoptera, Blattodea) with implications for taxonomy and evolutionary studies. European Journal of Taxonomy 291: 1-13. doi: 10.5852/ejt.2017.291

Legendre F, et al. 2015. Phylogeny of Dictyoptera: Dating the origin of cockroaches, praying mantises and termites with molecular data and controlled fossil evidence. PloS One 10: e0130127. doi: 10.1371/journal.pone.0130127

Li X, Wang Z 2015. A taxonomic study of the beetle cockroaches (Diploptera Saussure) from China, with notes on the genus and species worldwide (Blattodea: Blaberidae: Diplopterinae). Zootaxa 4018: 35-56. doi: 10.11646/zootaxa.4018.1.2

Liu JL, et al. 2023. Advances in the understanding of Blattodea evolution: insights from Phylotranscriptomics and Spermathecae. Molecular Phylogenetics and Evolution: 107753. doi: 10.1016/j.ympev.2023.107753

McKittrick FA 1964. Evolutionary studies of cockroaches. Cornell Experiment Station Memoir 389: 1-197.

Murienne J 2009. Molecular data confirm family status for the *Tryonicus*–*Lauraesilpha* group (Insecta: Blattodea: Tryonicidae). Organisms Diversity & Evolution 9: 44-51. doi: 10.1016/j.ode.2008.10.005

Risso VA, et al. 2015. Mutational studies on resurrected ancestral proteins reveal conservation of site-specific amino acid preferences throughout evolutionary history. Molecular Biology and Evolution 32: 440-455. doi: 10.1093/molbev/msu312

Roth LM 1995. *Africalolampra ehrmanni* New Genus and Species and the Male of *Paraplecta parva* Princis (Blattaria: Blaberidae). Psyche 102: 89-98.

Roth LM 1992. The Australian cockroach genus Laxta Walker (Dictyoptera: Blattaria: Blaberidae). Invertebr. Taxon. 6: 389-435.

Roth LM 1973a. The male genitalia of Blattaria XI. Perisphaeriinae. Psyche 80: 305-348.

Roth LM 1970a. The male genitalia of Blattaria. II. *Poeciloderrhis spp.* (Blaberidae: Epilamprinae). Psyche 77: 104-119.

Roth LM 1970b. The male genitalia of Blattaria. IV. Blaberidae: Blaberinae. Psyche 77: 308-342.

Roth LM 1970c. The male genitalia of Blattaria. V. Epilampra spp. (Blaberidae: Epilamprinae). Psyche 77: 436-486.

Roth LM 1971a. The male genitalia of Blattaria. VI Blaberidae: Oxyhaloinae. Psyche: 85-106.

Roth LM 1971b. The male genitalia of Blattaria. VII. *Galiblatta, Dryadoblatta, Poroblatta, Colapteroblatta, Nauclidas, Notolampra, Litopeltis,* and *Cariacasia* (Blaberidae: Epilamprinae). Psyche 78: 180-192.

Roth LM 1973b. The male genitalia of Blattaria. X. Blaberidae. *Pycnoscelus, Stilpnoblatta, Proscratea* (Pycnoscelinae), and *Diploptera* (Diplopterinae). Psyche 80: 249-264.

Roth LM 2003. Systematics And Phylogeny Of Cockroaches (Dictyoptera: Blattaria). Oriental Insects 37: 1-186.

Schneider A, Cannarozzi GM, Gonnet GH 2005. Empirical codon substitution matrix. BMC Bioinformatics 6: 134. doi: 10.1186/1471-2105-6-134

Sealfon RS, et al. 2015. FRESCo: finding regions of excess synonymous constraint in diverse viruses. Genome Biology 16: 38. doi: 10.1186/s13059-015-0603-7

Shen XX, Hittinger CT, Rokas A 2017. Contentious relationships in phylogenomic studies can be driven by a handful of genes. Nat. Ecol. Evol. 1: 126. doi: 10.1038/s41559-017-0126

Stamatakis A 2014. RAxML version 8: a tool for phylogenetic analysis and post-analysis of large phylogenies. Bioinformatics 30: 1312-1313. doi: 10.1093/bioinformatics/btu033

Tavaré S, Miura RM 1986. Some probabilistic and statistical problems in the analysis of DNA sequences. Lectures Math. Life Sci. 17: 57-86.

Usmanova DR, Ferretti L, Povolotskaya IS, Vlasov PK, Kondrashov FA 2015. A model of substitution trajectories in sequence space and long-term protein evolution. Molecular Biology and Evolution 32: 542-554. doi: 10.1093/molbev/msu318

Wang HC, Susko E, Roger AJ 2014. An amino acid substitution-selection model adjusts residue fitness to improve phylogenetic estimation. Molecular Biology and Evolution 31: 779-792. doi: 10.1093/molbev/msu044

Wang Y-S, et al. 2023. Phylogenetic analysis of Blaberoidea reveals non-monophyly of taxa and supports the creation of multiple new subfamilies. Cladistics. doi: <https://doi.org/10.1111/cla.12535>
